# Benchmarking MicrobIEM – a user-friendly tool for decontamination of microbiome sequencing data

**DOI:** 10.1186/s12915-023-01737-5

**Published:** 2023-11-23

**Authors:** Claudia Hülpüsch, Luise Rauer, Thomas Nussbaumer, Vera Schwierzeck, Madhumita Bhattacharyya, Veronika Erhart, Claudia Traidl-Hoffmann, Matthias Reiger, Avidan U. Neumann

**Affiliations:** 1 Environmental Medicine, Faculty of Medicine, University of Augsburg, Stenglinstr. 2, 86156 Augsburg, Germany; 2 Chair of Environmental Medicine, Technical University of Munich, Munich, Germany; 3 CK CARE, Christine Kühne Center for Allergy Research and Education, Davos, Switzerland; 4 Institute of Environmental Medicine, Helmholtz Munich, Augsburg, Germany; 5 Institute of Hygiene, University Hospital Muenster, Muenster, Germany; 6 ZIEL - Institute for Food & Health, Technical University of Munich, Freising-Weihenstephan, Germany

**Keywords:** Low-biomass microbiome, 16S rRNA gene sequencing, Bioinformatic decontamination, Negative control, Youden’s index, Decontam, SourceTracker

## Abstract

**Background:**

Microbiome analysis is becoming a standard component in many scientific studies, but also requires extensive quality control of the 16S rRNA gene sequencing data prior to analysis. In particular, when investigating low-biomass microbial environments such as human skin, contaminants distort the true microbiome sample composition and need to be removed bioinformatically. We introduce MicrobIEM, a novel tool to bioinformatically remove contaminants using negative controls.

**Results:**

We benchmarked MicrobIEM against five established decontamination approaches in four 16S rRNA amplicon sequencing datasets: three serially diluted mock communities (10^8^–10^3^ cells, 0.4–80% contamination) with even or staggered taxon compositions and a skin microbiome dataset. Results depended strongly on user-selected algorithm parameters. Overall, sample-based algorithms separated mock and contaminant sequences best in the even mock, whereas control-based algorithms performed better in the two staggered mocks, particularly in low-biomass samples (≤ 10^6^ cells). We show that a correct decontamination benchmarking requires realistic staggered mock communities and unbiased evaluation measures such as Youden’s index. In the skin dataset, the Decontam prevalence filter and MicrobIEM’s ratio filter effectively reduced common contaminants while keeping skin-associated genera.

**Conclusions:**

MicrobIEM’s ratio filter for decontamination performs better or as good as established bioinformatic decontamination tools. In contrast to established tools, MicrobIEM additionally provides interactive plots and supports selecting appropriate filtering parameters via a user-friendly graphical user interface. Therefore, MicrobIEM is the first quality control tool for microbiome experts without coding experience.

**Supplementary Information:**

The online version contains supplementary material available at 10.1186/s12915-023-01737-5.

## Background

Next-generation sequencing of microbial communities has revealed strong associations between human health and the microbiome [1–4]. However, biologically relevant differences in the microbiome between study groups [5–9] can be disguised by a range of biases [10–14].

One of these biases are contaminants, i.e., DNA sequences that are not truly present in the original microbial community [11, 15–19] and originate from external sources like extraction kits, lab consumables, or operators [16, 17]. Analyses of microbial mock communities and environmental microbiome samples have shown that contaminations are particularly problematic for low-biomass microbiome samples, where contaminants can make up the majority of detected sequences [20]. Consequently, samples with low microbial biomass such as from skin or lung can be biased by contamination and require appropriate tracking and identification of contaminants for the correct interpretation of microbiome data [21, 22].

Even though contaminants can be reduced by good laboratory practice [16], bioinformatic approaches are necessary for contaminant removal in low-biomass samples. Established approaches for bioinformatic decontamination can be broadly placed into three categories: blacklist-based approaches (i) remove contaminants based on established lists of common contaminants [23, 24], independent of the sampled environment. Sample-based decontamination approaches (ii) do not require negative controls for removing contaminants, but identify contaminants based on their relative abundance in the samples. This is implemented, for example, in the frequency filter of the tool Decontam, where contaminants are identified based on the negative correlation between their relative abundance and total DNA per sample [25]. Control-based approaches (iii) require negative controls to be processed along with the samples. Typical controls comprise pipeline negative controls, which gather contaminants over the complete data generation pipeline, and PCR controls, which are added prior to the PCR amplification and can therefore only cover contaminants introduced from the PCR onwards [26]. Using these controls, contamination removal can be as simple as removing every sequence from a dataset that appears in the negative controls, or using more complex algorithms like the Decontam prevalence filter or SourceTracker [25, 27].

In this manuscript, we introduce our novel control-based decontamination tool MicrobIEM, which identifies contaminants based on their relative abundance in negative controls compared to environmental samples and their consistent occurrence in negative controls. In contrast to other established decontamination tools, our algorithm can be used either script-based or through a graphical user interface with interactive plots to visualize taxa in negative controls. Thus, MicrobIEM addresses the growing need for microbiome quality control tools suitable for scientists without coding experience.

To benchmark the efficiency of these decontamination tools and their tool-specific filter parameters, mock communities with known sequence composition can be used to discriminate between true sequences and contaminants. Previously, bioinformatic decontamination tools were benchmarked using a whole-cell, evenly composed mock community [20] in a dilution series to cover the expected biomass of the environmental microbiome samples under investigation. While this groundbreaking work of Karstens et al. has significantly advanced the field and has guided numerous microbiome researchers in recent years, additional aspects may need to be considered when benchmarking decontamination approaches. In particular, mock communities with an even taxon composition may not sufficiently represent natural complex microbiome communities [28–30], which may be better represented by a staggered mock composition. Additionally, to quantify the decontamination success of each tool, the choice of an appropriate evaluation score for the benchmarking is crucial. Building upon knowledge from the field of machine learning, the more unbiased scores Youden’s index and Matthews correlation coefficient can supplement the traditionally used accuracy [31, 32].

In this paper, we benchmarked established decontamination approaches and our novel tool MicrobIEM in an even mock community and two staggered mock communities. We focused on Youden’s index as an evaluation score to identify effective bioinformatic decontamination approaches for amplicon sequencing data. Furthermore, we show the effect of these different decontamination tools on a low-biomass skin microbiome dataset.

## Methods

### Study design

**Fig. 1:**
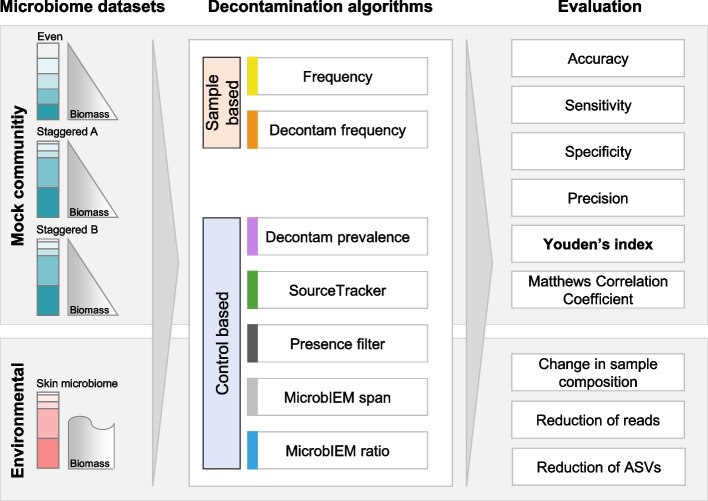
Overview of the decontamination benchmarking study design. Three mock datasets were used for decontamination benchmarking, one with an even, and two with a staggered community structure. Mock communities were available as dilution series covering a wide range of bacterial biomass per sample (10^8^ to 5.55 × 10^3^ bacterial cells). Two sample-based and five control-based decontamination algorithms were compared based on their classification performance into mock and contaminant reads, evaluated by Youden’s index and other evaluation scores. The same parameters and tools were also evaluated in a low-biomass environmental dataset from the skin. Additional information about the decontamination filters implemented in MicrobIEM can be found in Additional file 1: Supplementary Figure 1

An overview of the study design is shown in Fig. 1. We benchmarked six decontamination approaches and their parameters in three mock communities with different sample compositions, and in an environmental low-biomass microbiome dataset. Each mock dataset was available as a dilution series, where we used samples ranging from high bacterial input (10^8^ cells) to low bacterial input (10^3^ cells) in the benchmarking to mimic different microbial source environments. The chosen decontamination algorithms were either sample based (frequency filter, Decontam frequency filter) or control based (presence filter, Decontam prevalence filter, SourceTracker, and the ratio and span filter of our novel tool MicrobIEM). For each algorithm, we also tested tool-specific parameters for contaminant removal, which have to be selected by the user. To evaluate the success of each decontamination approach, we compared common test assessment scores for each dilution of the mock communities. Additionally, the effect of the decontamination algorithms on the sample composition and number of taxa and reads was investigated in an environmental low-biomass dataset.

### Datasets for benchmarking

The datasets consisting of three mock communities and one environmental low-biomass microbiome dataset are described below and an overview is provided in Additional file 1: Supplementary table 1.

#### Even mock community

As an even mock community, we used the dataset of a previous decontamination benchmarking of Karstens et al. [20]. The complete data preparation methods can be found in the original publication. Briefly, the Zymobiomics D6300 mock community (https://www.zymoresearch.de/collections/zymobiomics-microbial-community-standards/products/zymobiomics-microbial-community-standard) consists of eight bacterial and two fungal species in an even composition in terms of total DNA, with proportions of cell numbers per bacterial species ranging from 6 to 22%. Of this mock community, a serial threefold dilution was prepared, from 1.5 × 10^9^ to 2.3 × 10^5^ input cells. Additionally, one pipeline negative control is available. The V4 region of the bacterial 16S rRNA gene was amplified and sequenced on an Illumina MiSeq® platform (Illumina Inc.). Reads were denoised using DADA2 [33] and annotated using the Silva database [34]. Mock ASVs were defined based on ASVs present in the undiluted sample, with sequences classified as mock that matched the expected 16S reference sequences by Zymobiomics exactly or with one nucleotide difference. All other ASVs in the undiluted sample differed substantially from any expected sequence, as described by Karstens et al. [20] (https://github.com/lakarstens/ControllingContaminants16S). All remaining non-mock ASVs present in the data were classified as contaminants. The final dataset consists of 1,675,028 reads (median 172,915 reads per mock sample, 189,779 reads in the negative control) in 1414 ASVs, of which 9 ASVs were classified as mock and 1405 as contaminants. The ASV table and contaminant classification were taken as submitted by the original authors on Github [https://github.com/lakarstens/ControllingContaminants16S].

#### Staggered mock community A

To also test decontamination approaches in a more realistic, uneven community structure [28–30], we created a staggered mock community called “A”, consisting of 15 strains that differ in their absolute cell counts by two orders of magnitude from 18 to 0.18% (Additional file 1: Supplementary table 2). From this staggered mock community, we prepared a serial tenfold dilution from 10^9^ to 10^2^ cells in three technical replicates per dilution. Additionally, three pipeline negative controls and three PCR controls were processed.

##### Mock preparation

Aerobic and anaerobic bacteria of the mock community were cultivated with the appropriate medium, temperature and oxygen supply at each of the following steps as summarized in Additional file 1: Supplementary Table 2. Pre-cultures were obtained by inoculating 3 mL of medium, which were then grown for 6 h. Next, 10 μL of the pre-culture was transferred to flasks (baffled flask if oxygen required for growth) containing either 100, 250 or 500 mL of the respective medium depending on their growth capacity. Overnight cultures were centrifuged for 10 min at 3000 × *g* and washed 3 times. Each culture was aliquoted into one part for storage at − 80 °C and one part for cell number determination. To ascertain the cell number, the optical density (OD) was measured and an OD_600_ of 1 was equated with 10^9^ cells. Subsequently, a dilution series was prepared, and 50 μL of the dilutions expecting 1, 10, 100, and 1000 cells per plate was plated with a Drigalski spatula. Colony-forming units were counted to determine the cell number. Lastly, the required amount of the strains was mixed to obtain the desired cell number for each strain of the mock community with the composition as described in Additional file 1: Supplementary Table 2.

##### DNA extraction and sequencing

The microbial DNA of all samples and the three pipeline negative controls was extracted with the UCP Pathogen Kit (Qiagen) according to the manufacturer’s instructions in an elution volume of 80 μL. Cell lysis was performed in screw cap tubes containing the sample, 500 mg of 100 μm diameter zirconia-silica beads, 500 μL Stool Stabilizer (Stratec), and 650 μL of ATL buffer containing 4.3 μL DX buffer, with a Precellys Evolution device (Bertin) shaking twice for 90 s with a 15-s break. The V1–V3 variable region of the 16S rRNA gene was amplified using the primers 27F-YM (5'-AGAGTTTGATYMTGGCTCAG-3') and 534R (5'-ATTACCGCGGCTGCTGG-3') with the Q5 High-Fidelity PCR kit (New England Biolabs) with the following conditions: 98 °C for 10 s, 59 °C for 20 s, 72 °C for 15 s for 25 cycles. In a subsequent 8-cycles PCR reaction, barcodes for all samples and PCR controls were added. Indexed amplicons were purified using AMPure XP beads (Beckman Coulter) with a bead to DNA ratio of 0.7:1 (vol/vol), according to the manufacturer’s instructions. The purified amplicons were quantified with the fluorescent dye-based Qubit® dsDNA HS Assay Kit (Invitrogen) and all samples were pooled equimolarly. Sequencing was carried out on an Illumina MiSeq® platform (Illumina Inc.) using 2 × 300 bp paired-end reads at the Core Facility Microbiome at ZIEL, Institute for Food and Health, Freising, Germany.

##### Bioinformatic processing of samples

The sequences were denoised using DADA2 [33] with default parameters except truncLen = c(299,280), trimLeft = c(20,17), and maxEE = c(2,6) in the function filterAndTrim(), and annotated using RDP-based annotation formatted for DADA2 [35]. Sequences which differed at least 20% from the expected sequence length were removed as well as singletons. One sample was removed due to experimental failure (10^3^ input cells, 11 reads), leading to a total of 23 mock samples for decontamination analysis. Mock ASVs were defined as sequences matching a 362-bp-long subsection of the V1–V3 region of the expected sequences based on Sanger sequencing of individual mock taxa. Since Sanger sequencing produces only one read-out even in the case of different 16S copy variants per species, we tolerated ambiguous base calls and additionally accepted ASVs differing by up to 4 bp (Levenshtein distance) from expected ASVs. The final dataset of the “staggered mock A” consists of 361,651 reads (median 13,747 reads per mock sample, median 1226 reads in the negative controls) in 293 ASVs, of which 52 ASVs were classified as mock and 241 as contaminants.

#### Staggered mock community B

To validate our analyses in a second realistic mock community with an uneven community structure, we used a subset of the dataset published by Rauer & de Tomassi et al. [36]. The complete experimental design and data preparation methods can be found in the corresponding manuscript. Briefly, this study compared eight extraction protocols in three mock communities. Here, we used only a subset of eight samples of the three-species spike-in mock community D6321 (https://www.zymoresearch.de/collections/zymobiomics-microbial-community-standards/products/zymobiomics-spike-in-control-ii-low-microbial-load), which were processed using the ZymoResearch extraction buffer. The undiluted samples with 1.1 × 10^5^ bacterial input cells were 1:20 diluted to 5.55 × 10^3^ bacterial input cells, with each of the two dilutions present in four replicates. These replicates underwent different extraction kits and protocols, but shared the same extraction buffer that was associated with low-level contamination. Additionally, four pipeline negative controls (using the same extraction buffer) and two PCR controls were processed along with the samples. The V1–V3 region of the 16S rRNA gene was sequenced using the Illumina MiSeq® platform (Illumina Inc.). Mock ASVs were defined as sequences with Levenshtein distance ≤ 4 to any of the three reference 16S genes provided by ZymoResearch. We selected this subset of samples due to the presence of cross-contaminating mock ASVs in the negative controls, and refer to the dataset here as “staggered mock community B”. The staggered mock B had 195,279 reads (median 25,282 per mock sample, median 6616 reads in the pipeline negative controls). Of all 221 ASVs, 9 ASVs were classified as mock and 212 as contaminants.

#### Skin microbiome dataset

As a low-biomass environmental microbiome dataset, we chose a skin microbiome dataset published by Hülpüsch et al. [37]. The skin is a low-biomass environment (10^3^ to 10^7^ bacteria [38, 39]) and is therefore representative for an environment susceptible to contaminants. Via qPCR, a bacterial colonization of 10^6^ cells was determined as published by De Tomassi et al. [40]. The longitudinal study investigated the skin microbiome of six healthy individuals and six atopic eczema (atopic dermatitis, AD) patients over the course of 8 weeks. The study was approved by the ethics committee of the Technical University of Munich (187/17S). Complete methods for data generation can be found in the original publication. Briefly, Illumina MiSeq® sequencing (Illumina Inc.) of the V1–V3 16S rRNA gene was performed. In contrast to the previous publication, the sequences were now denoised using DADA2 [33] with default parameters except truncLen = c(299,280), trimLeft = c(20,17), and maxEE = c(2,6) in the function filterAndTrim(), and annotated using AnnotIEM [41]. In total, 209 samples and 12 pipeline negative controls were analyzed (sequencing depth of samples > 5000 reads). The skin microbiome dataset consists of 4,836,304 reads and 21,541 ASVs.

### Contaminant removal approaches for benchmarking

In the following section, we describe in detail the chosen decontamination tools used for the benchmarking. As the blacklist approach does not consider sample-specific environments, it is not recommended [20] and was not considered in this benchmarking.

#### Frequency filter

A frequency filter removes all sequences from a dataset, which do not reach a certain threshold of relative abundance in any sample. The rationale behind this method is that singletons and sequences with low relative abundance often represent incorrect sequences like chimeras, so their removal from microbiome datasets decreases computational times and reduces differences between biological and technical replicates and is therefore advisable [42, 43]. There is no commonly accepted threshold, but values of 0.00005 (0.005%) [44] or 0.0025 (0.25%) [45] have been recommended.

#### Presence filter

The presence filter removes every sequence which appears in any negative control [20] without considering its abundance or taxonomic annotation.

#### SourceTracker

SourceTracker is a Bayesian approach to model the sources and proportions of contaminants in microbiome studies. It was published by Knights et al. in 2011 [27] and is available as an R script (version 1.0.1, https://github.com/danknights/sourcetracker). In contrast to other tools, SourceTracker classifies individual reads of a sequence, allowing a sequence to originate from different sources. Source environments can be the “unknown” biological sample, or defined external sources (e.g., laboratory bench, reagents). Thus, SourceTracker can use negative controls as a single external source environment, but works best with additional samples from the environment [27] or knowledge on the sampled communities [20]. SourceTracker has previously achieved great decontamination results when this additional knowledge on the sampled communities or additional types of controls were incorporated [20]. However, as this data is often not available in clinical microbiome studies, we decided to benchmark only the most often encountered scenario in microbiome research, where prior knowledge on expected and contaminant taxa is not reliably available. We therefore only use the pipeline negative controls as source environments per dataset here. The three parameters alpha1 (default 0.001), alpha2 (default 0.1), and beta (default 10) can be adapted to avoid overfitting of the data and increase the sensitivity of the algorithm.

#### Decontam

Decontam identifies contaminants by a sample-based and a control-based algorithm, which can be used individually or combined. The method is available as an R package (version 1.8.0) and has been published by Davis et al. in 2018 [25].

##### Decontam frequency filter

The Decontam frequency filter is based on the hypothesis that the relative abundance of contaminating sequences is inversely correlated with the total input material of a sample. Therefore, additional data on DNA concentration is required, such as measured via qPCR or fluorescence dye-based methods. The user-set threshold ranges from 0 to 1 (default 0.1) and can be increased to achieve a stricter contaminant removal.

##### Decontam prevalence filter

The Decontam prevalence filter is based on negative controls. For each sequence, the presence or absence in controls is compared to environmental samples. Again, the threshold ranges from 0 to 1 (default 0.1) and can be increased to achieve a stricter contaminant removal.

#### MicrobIEM

MicrobIEM is our novel tool implemented in the statistical software package R [46], providing decontamination, quality control and basic analysis of microbiome (amplicon sequencing) samples. The decontamination core algorithm is implemented as a single R function, and as a complete tool with graphical user interface and basic analysis options for researchers without coding experience. The complete code and user documentation are provided in the Github repository (https://github.com/LuiseRauer/MicrobIEM), and the tool can be used directly through a web browser without installation (https://env-med.shinyapps.io/microbiem/).

##### Decontamination with MicrobIEM ratio filter and span filter

In MicrobIEM, contaminant removal is based on negative controls (Additional file 1: Supplementary Figure 1). As identifying contaminant features solely by their presence in negative controls is not recommended [20], we developed two new concepts: (1) the ratio of the mean relative abundance of a sequence in negative controls versus in environmental samples, since a “systematic” contaminant (i.e., not sporadic, originating from, e.g., lab consumables) should appear in rather high relative abundance in the expectably empty negative controls (with contaminants defined as: (mean relative abundance of feature in control / mean relative abundance of feature in sample) > threshold), and (2) a span threshold measuring the proportion of negative control samples contaminated with this sequence, since “systematic” contaminants should not appear only sporadically in a small fraction of negative controls (with contaminants defined as (span in controls ≥ threshold)). Therefore, MicrobIEM’s span filter with a threshold of 1 (out of all available controls) is identical to the presence filter, while increasing the span threshold is intended to alleviate the effect of sporadic cross-contamination from samples into negative controls. The number of available thresholds for MicrobIEM’s span filter depends on the number of control samples per dataset. MicrobIEM’s two contamination filters can be applied independently for two types of controls (e.g., PCR controls and pipeline controls as in our dataset). In our benchmarking, we only test MicrobIEM’s two filters on pipeline negative controls, since barely any reads were detected in the available PCR controls.

##### Additional quality control & data analysis options in MicrobIEM

The decontamination algorithm, additional quality control options, and basic statistical analyses are implemented in a Shiny-based tool with a graphical user interface and interactive plots created with plotly [47, 48]. Thus, researchers without coding experience are able to inspect and explore microbiome data easily, fast, and interactively. An overview of the available filter steps and workflow is given in Additional file 1: Supplementary Figure 2. As input, a feature table with read counts (either ASV or operational taxonomic unit (OTU) table) and a metadata table with additional information per sample (e.g., gender, treatment) and a specification as “sample” or “NEG1”/“NEG2” (controls) is required. Quality control options in MicrobIEM (Additional file 1: Supplementary Figure 2B) include the removal of samples with a low number of total reads, as these samples often represent experimental failure due to technical problems in sampling or amplification [49]. Additionally, features with a low maximum abundance over all samples (e.g., singletons or doubletons) or with a low relative abundance over all samples (equivalent to the frequency filter) can be removed as they often represent spurious sequencing errors or contaminations [45]. All quality control steps can be used independently or in combination. Thresholds for these steps are defined by the user, but their impact on the data can be assessed with the interactive plots offered in MicrobIEM.

After quality control, the final filtered feature table can be explored and visualized interactively with the most common analyses for microbiome research, namely alpha diversity, beta diversity, and the distribution of microbial taxa (taxonomic composition). Two-sided statistical tests (Kruskal–Wallis and Permutational Multivariate Analysis of Variance, PERMANOVA) are provided for alpha and beta diversity analyses, respectively. More details on the statistical analysis in MicrobIEM are provided in the readme on Github (https://github.com/LuiseRauer/MicrobIEM).

##### Example dataset

For an initial exploration of the functions of MicrobIEM, we provide an artificial example dataset, consisting of a feature table (MicrobIEM_test-data_featurefile.txt) and a metadata table (MicrobIEM_test-data_metafile.txt). The dataset contains 29 microbiome samples that can be investigated by age group, gender, BMI, and medication dosage. Additionally, we provide 1 positive control, and 2 types of negative controls: 3 PCR controls (NEG1) and 2 pipeline controls (NEG2). The example datasets are deposited on Github (https://github.com/LuiseRauer/MicrobIEM/tree/main/MicrobIEM/test-data) and on the Open Science Framework platform (https://osf.io/xvbef/).

### Benchmarking evaluation measures

#### Diagnostic power in mock communities

To evaluate the effect of different decontamination tools in mock community datasets, we compared a tool’s classification into mock or contaminant reads with the true classification, given by the expected sequences per mock. We evaluated accuracy, sensitivity, specificity, precision, Matthews correlation coefficient, and Youden’s index.

Here, sensitivity (also called recall) is the ability of each algorithm to correctly remove a contaminant ASV, and specificity is the ability of each algorithm to correctly keep an expected mock ASV in the dataset. Accuracy measures the proportion of correct classifications among all classifications and is a commonly used evaluation index. However, it can give misleading results in case of class imbalance, i.e., when samples have very high or very low proportions of contaminants. Additionally, accuracy needs to be interpreted in relation to baseline accuracy, which corresponds to contaminant prevalence in our samples. The frequently recommended Matthews correlation coefficient (MCC) quantifies the correlation between the true classification and the tool’s classification and gives fairer results by incorporating information on both correct and incorrect classifications. MCC is also affected by class imbalance and is thus not comparable between studies or samples with different proportions of contaminants. Youden’s index (also called bookmaker informedness) incorporates sensitivity and specificity. It is not affected by class imbalance and is therefore suitable for all levels of contaminant prevalence and comparable between samples and datasets. Precision measures the proportion of correctly removed contaminant ASVs among all removed ASVs.

Sensitivity, specificity, accuracy, and precision range from 0 to 1, with 0.5 indicating a classification as good as random (0 in case of precision). Youden’s index and MCC range from − 1 to 1, with 0 indicating a classification as good as random, and negative values indicating a deterioration compared to the original composition due to reversed labels. For all evaluation measures, 1 indicates a perfect classification.

#### Evaluation of the skin microbiome dataset

We evaluated the sample composition of the 10 most abundant genera before and after applying the decontamination algorithms, as well as the reduction in reads and number of ASVs. Since the expected sequences are not known in the skin microbiome dataset, we instead evaluated the removed and kept genera per decontamination tool and filter setting. Therefore, lists of typical contaminants and typical skin inhabitants were created at the genus level according to a literature search (Additional file 1: Supplementary Table 3, 4 and 5). Typical contaminants were defined based on ten papers dealing with contaminants (Additional file 1: Supplementary Table 3) [15, 16, 18, 22, 23, 50–54]. To determine skin bacteria, seven papers were selected for the classification, representing a mix of skin microbiome-related original publications, reviews, sequencing studies, and cultivation studies (Additional file 1: Supplementary Table 4) [55–61]. The occurrences of each skin genus in the seven papers are summarized in Additional file 1: Supplementary Table 5.

### Statistical analysis

Benchmarking analysis was performed in R 4.0.2 [46], and MicrobIEM analysis of the skin microbiome dataset was performed in version 0.7 (https://github.com/LuiseRauer/MicrobIEM). Analysis scripts are available on Github for benchmarking of the mock communities (https://github.com/LuiseRauer/Decontamination_benchmarking) and at the Open Science Framework (OSF) platform for benchmarking of the skin microbiome dataset (https://osf.io/yn9sa/).

## Results

### Susceptibility of low-biomass samples to contaminants

We investigated serial dilutions of three mock communities, one with an even composition (6–22% relative abundance per strain), and two with a staggered composition (A: 0.18–18% relative abundance per strain, B: 3–84.3% relative abundance per strain) were used.

**Fig. 2:**
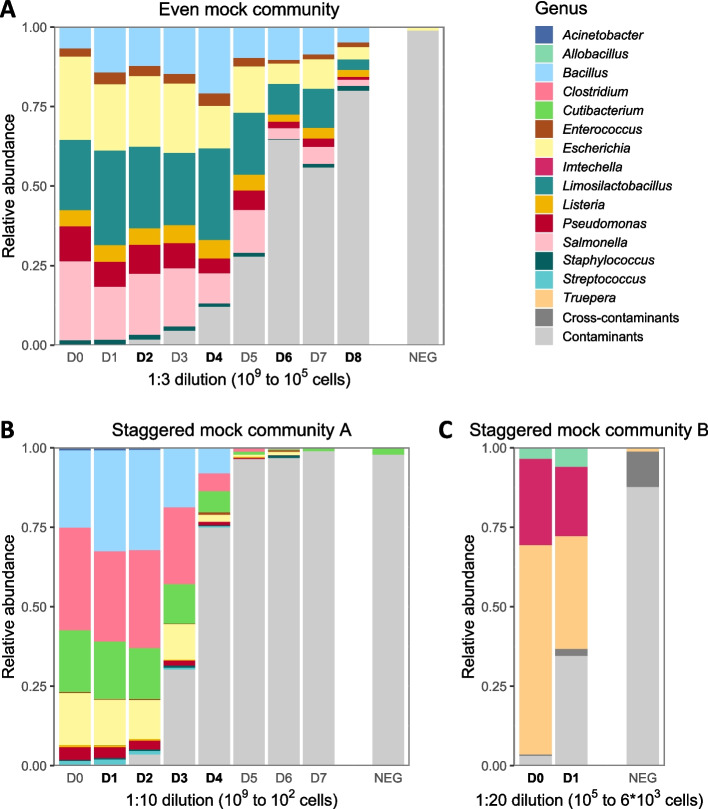
Sample composition and level of contamination by dilution in the mock communities used for benchmarking. The proportion of contaminants increases with decreasing amount of bacterial input material, both in the even mock community (**A**) and in the two staggered mock communities A and B (**B**, **C**). The even mock community (**A**) contains species of 6–22% expected relative abundance, and comprises threefold serial dilutions from 1.5 × 10^9^ to 2.3 × 10^5^ bacterial input cells and one pipeline negative control (NEG). The staggered mock community A (**B**) contains species of 0.18–18% expected relative abundance, and comprises tenfold serial dilutions from 1 × 10^9^ to 1 × 10^2^ bacterial input cells and three pipeline negative controls (NEG). The staggered mock community B (**C**) contains species of 3–84.3% expected relative abundance, and comprises 20-fold serial dilutions from 1.1 × 10^5^ to 5.55 × 10^3^ bacterial input cells and four pipeline negative controls (NEG). Each bar in **B** shows the mean composition per triplicate (10^3^: duplicate) per dilution, and each bar in **C** shows the mean composition over four replicates per dilution. Reads not matching expected sequences were defined as contaminants (see details in “Methods”). Dilutions highlighted in bold are selected for decontamination benchmarking

In all three mock community datasets, samples with lower-input material were characterized by higher proportions of contaminants, as defined by sequences not matching the expected mock sequences (Fig. 2A, B). The even and the staggered dataset A contained similar proportions of contaminants per amount of input material, with less than 2% contaminants in samples with high-input material (10^8^–10^9^ cells) and more than 50% contaminants in samples with low-input material (< 10^6^ cells). In samples with input material of less than 10^4^ cells in the staggered mock community A (Fig. 2B), more than 96% of sequences were contaminants, similar to the negative controls. The staggered mock community B (Fig. 2C) was slightly less contaminated, with only 34.5% of contaminant reads (median) in samples with 5.55 × 10^3^ input cells. Of note, the negative controls of all three mock datasets contained only minor proportions of expected mock taxa (< 5%), belonging to *Escherichia/Shigella*, *Enterococcus*, or *Staphylococcus* in the even mock, to *Cutibacterium* in the staggered mock A, and to *Truepera* and *Imtechella* in the staggered mock B. In the staggered mock A, two pipeline controls contained only contaminant taxa, while only one out of the three available pipeline controls contained any mock sequences (1 ASV). In the staggered mock community B, we detected more cross-contamination into negative controls, with mock taxa present in two out of four negative controls (1 and 2 ASVs, respectively).

### Benchmarking of decontamination algorithms in mock communities

**Fig. 3:**
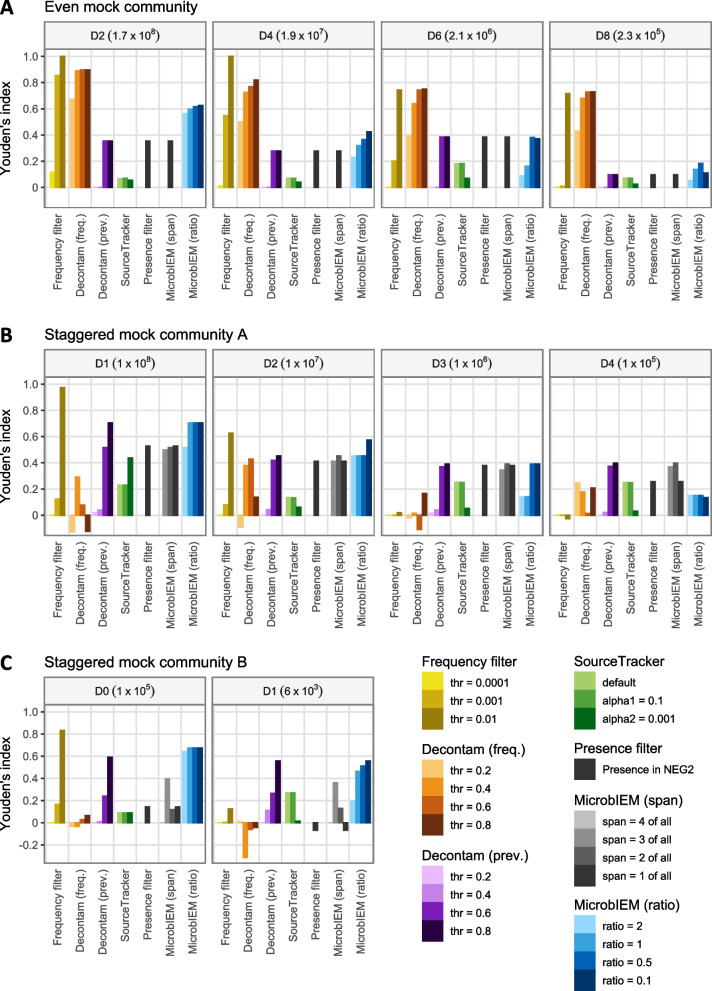
Benchmarking of decontamination algorithms in mock communities. In the even mock community (**A**), sample-based decontamination algorithms perform best (frequency filter, Decontam frequency filter); whereas in the staggered mock communities A and B (**B**, **C**), control-based decontamination algorithms perform better (Decontam prevalence filter, SourceTracker, presence filter, MicrobIEM span filter, MicrobIEM ratio filter). MicrobIEM’s span filter of “1 of all” is equivalent to the presence filter, and the number of available thresholds for MicrobIEM’s span filter depends on the number of negative controls per dataset (**A**: 1, **B**: 3, **C**: 4 pipeline negative controls). Each algorithm was evaluated by its ability to distinguish expected mock reads from contaminating reads (defined by reads not matching expected sequences), from high (10^8^) to low-biomass samples (10^3^ bacterial cells). The performance per algorithm was quantified by Youden’s index, ranging from 1 (perfect classification) over 0 (random classification) to − 1 (indicating reversed labels). Algorithms were run separately per dilution, except for the Decontam frequency filter in **A** and SourceTracker in all datasets. Values in **B** represent mean values over triplicates per dilution, and values in **C** represent mean values over four replicates per dilution. Freq. = frequency, prev. = prevalence

The effects of six different decontamination algorithms and their tool-specific parameters was analyzed on the even and the two staggered mock communities (Fig. 3). For benchmarking, we focused on samples with an input material between 10^5^ and 10^8^ cells input material for the even and the staggered mock A, and on both dilutions of the staggered mock B between 10^5^ and 10^3^ bacterial input cells, jointly covering 0.4 to 80% contamination. Higher input material samples of the even and staggered mock A contained similar proportions of contaminants as the 10^8^ samples, whereas samples with lower-input material of the staggered mock A contained > 96% contamination and were therefore neglected.

#### Even mock community

In the even mock community (Fig. 3A), the sample-based decontamination algorithms (frequency filter and Decontam frequency filter) generally achieved the best decontamination classification in each dilution, indicated by the highest Youden’s index. However, this critically depended on the chosen thresholds: While a strict frequency filter of 0.01 distinguished perfectly between contaminants and mock reads in some dilutions, the relaxed threshold of 0.0001 performed very poorly. For the Decontam frequency filter, stricter thresholds of 0.8 or 0.6 achieved better classifications into mock and contaminant sequences, and the filter’s performance was reduced when decreasing the threshold to 0.2.

Comparing only the control-based decontamination algorithms, MicrobIEM’s ratio filter performed better or as good as the presence filter and the Decontam prevalence filter, depending on the amount of input material. Stricter MicrobIEM ratio filters of 0.1 and 0.5 were slightly more effective than more moderate ratios of 1 or 2. Since only one negative control was available in the even mock community, MicrobIEM’s span filter was identical to the presence filter. Similarly, the Decontam prevalence filter could hence not leverage its potential: a loose filter of 0.2 or 0.4 did not remove any contaminants, and the stricter thresholds of 0.6 and 0.8 removed all sequences present in the negative control, identical to the presence filter.

#### Staggered mock communities

In the staggered mock community A and B (Fig. 3B, C), all decontamination approaches could be benchmarked on triplicates and quadruplicates of samples and controls. Overall, sample-based decontamination algorithms reached lower Youden’s scores than in the even mock communities, and were thus outperformed by control-based methods, especially in low-biomass samples with higher proportions of contamination. While a strict frequency filter of 0.01 still achieved a convincing result in samples with less contamination (10^8^ input cells in staggered mock A, 10^5^ input cells in staggered mock B), its performance with other thresholds or in lower-input samples was reduced compared to the other decontamination approaches. Generally, control-based tools reached similar Youden’s scores in the even and the staggered mock communities. SourceTracker generally did not perform well in any of our mock communities; however, we did not benchmark all the functionalities of this powerful tool, which can be greatly improved by adding additional types of control samples not available in our design. Except for SourceTracker, the four other control-based algorithms performed very similar in the staggered mock A, with MicrobIEM’s ratio filter achieving the best classification in samples with medium levels of contamination (10^7^ input cells), and Decontam prevalence filter reaching the highest Youden’s score in the lowest-input samples. In the staggered mock community B, MicrobIEM’s ratio filter and Decontam’s prevalence filter outperformed other control-based decontamination algorithms. Interestingly, while the chosen threshold in MicrobIEM’s ratio filter did not drastically affect the results, stricter Decontam prevalence thresholds of 0.6 or 0.8 generally achieved better results. MicrobIEM’s span filter achieved medium results in both staggered mock communities. Generally, its performance was similar or improved compared to the presence filter in almost all dilutions, but particularly in the staggered mock B, which was characterized by increased cross-contamination into negative controls. A combination of MicrobIEM’s ratio and span filter did not notably improve the decontamination results in any of the staggered mocks and can therefore not be recommended (Additional file 1: Supplementary Figure 3).

In all three mock communities, better classification into mock and contaminants was achieved in samples with higher input material, and none of the algorithms performed very well in low-input samples, particularly in the staggered mock communities. However, MicrobIEM’s ratio filter and Decontam’s prevalence filter generally achieved relatively similar and consistently good results in the two staggered mock communities.

### Results of other evaluation measures

As an overall trend, all tools achieved higher specificity than sensitivity, independent of the amount of input cells and community structure (Additional file 1: Supplementary Figure 4A, B, C). Almost every tool had a filter setting with perfect specificity across all dilutions, thus keeping all mock ASVs. However, the tools varied greatly in their ability to remove contaminants (sensitivity). Best sensitivity values were generally achieved with stricter filter settings, as indicated by darker color, for example with a frequency filter of 0.01, Decontam prevalence/frequency filter of 0.8, or MicrobIEM’s ratio filter of 0.1. Confirming the well-known trade-off between sensitivity and specificity, these stricter thresholds however decreased specificity. Exceptions to that, with both high sensitivity and high specificity, were achieved by the sample-based tools in the even mock, and by the control-based tools Decontam prevalence and MicrobIEM ratio in the staggered mocks.

In general, the conservative behavior of classifying most features as mock reads leads to high accuracy values, even when a tool does not remove any contaminants. The case of all features being classified as mock reads can be measured by “baseline accuracy,” and corresponds to the contaminant prevalence per dilution. Supplementary Fig. 4 (Additional file 1) shows that the tools’ accuracy is identical or only slightly better than baseline accuracy in some cases. In contrast, Youden’s index and MCC deliver a more intuitive interpretation with good classification results indicated by values larger than 0, independent of the contaminant prevalence per dilution. The results of Youden’s index and MCC are very similar, except for a few cases, e.g., for the frequency filter of 0.01 in the staggered mock community (Additional file 1: Supplementary Figure 4B), where MCC reacts to the class imbalance in high-biomass samples. Precision-recall curves with additional filter thresholds per algorithm and dilution are given in Additional file 1: Supplementary Figure 5.

### Influence of decontamination algorithms in a low-biomass microbiome dataset

To show the decontamination effects on environmental microbiome data, we selected a skin microbiome dataset with approximately 10^6^ input cells (determined by qPCR). We applied all decontamination algorithms with all thresholds on the skin dataset like in the mock datasets. Since in the skin dataset, the distinction between contaminant and skin inhabitant is impossible, we analyzed the effect on the decontamination tools on the 10 most abundant genera. To evaluate the algorithm’s performances, a literature search was performed to define expected skin inhabitants or typical contaminants (Additional file 1: Supplementary Table 3, 4 and 5). Due to a large overlap between these two lists, a clear distinction was not always possible. Based on the literature search, the unfiltered skin microbiome dataset as well as the negative controls contained a mix of typical skin inhabitants, and typical contaminants, but the largest proportion comprised genera that appeared both as contaminants and typical skin inhabitant (e.g., *Corynebacterium*, *Propionibacterium*, *Staphylococcus*) (Additional file 1: Supplementary Figure 6). Although these three genera also appear as typical contaminants, their presence on the skin is well-documented by amplicon sequencing, metagenomics, and cultivation studies [55–61], and they were therefore considered as skin inhabitants in our skin microbiome dataset.

**Fig. 4:**
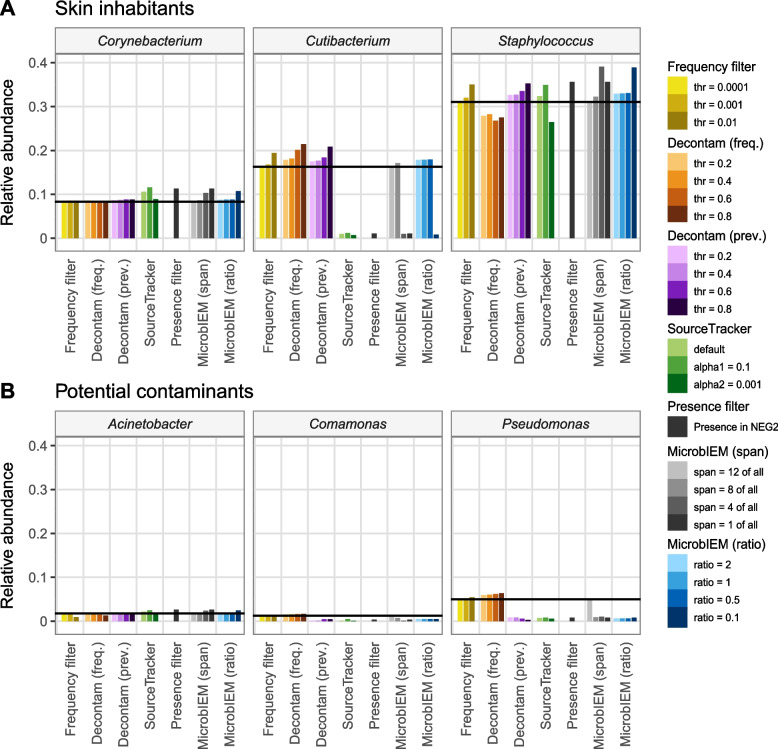
Effect of decontamination algorithms on major skin inhabitants and contaminants in a low-biomass skin microbiome dataset. The effect of six bioinformatic decontamination algorithms with tool-specific thresholds was evaluated on three typical skin inhabitants (*Corynebacterium*, *Cutibacterium*, and *Staphylococcus*) and three potential contaminants (*Acinetobacter, Comamonas, Pseudomonas*). While sample-based decontamination algorithms (frequency filter, Decontam frequency filter) had little effect on the relative abundance of the top 10 genera of the low-biomass skin microbiome dataset, control-based decontamination algorithms (Decontam prevalence filter, SourceTracker, presence filter, MicrobIEM span filter, MicrobIEM ratio filter) specifically reduced *Pseudomonas* and *Comamonas*. MicrobIEM’s span filter of “1 of all” is equivalent to the presence filter. Horizontal black lines indicate the relative abundance per genus before applying the bioinformatic decontamination approaches. Freq. = frequency, prev. = prevalence

Generally, sample-based decontamination algorithms (frequency filter, Decontam frequency filter) only slightly altered the sample composition of the top 10 genera compared to the unfiltered skin microbiome dataset (Fig. 4, Additional file 1: Supplementary Figure 6, 7). In contrast, the control-based decontamination algorithms strongly reduced specific taxa of the 10 most abundant genera. Interestingly, the presence filter, the strict MicrobIEM span filter and SourceTracker lead to a similar sample composition after filter application, while also the Decontam prevalence filter and MicrobIEM ratio filter performed similarly.

The relative abundance of the typical skin inhabitants *Corynebacterium* and *Staphylococcus* was not significantly altered by the majority of decontamination algorithms and thresholds, also in relation to the original relative abundance (Fig. 4A), even though these skin species appeared in the pipeline negative controls. Contrarily, the presence filter and SourceTracker both strongly reduced the relative abundance of the typical major skin inhabitant *Cutibacterium* from the skin dataset independent of the threshold used, since these reads occurred in low levels in the pipeline negative controls (Fig. 4, Additional file 1: Supplementary Figure 6). In contrast, *Cutibacterium* was kept by the Decontam prevalence filter and the MicrobIEM ratio filter. The strict MicrobIEM span filter performed like the presence filter, while less strict thresholds were similar to the MicrobIEM ratio filter and Decontam.

All control-based decontamination algorithms (presence filter, Decontam prevalence, SourceTracker and MicrobIEM ratio and span filter) reduced the relative abundance of the potential contaminants *Pseudomonas* and *Comamonas* (Fig. 4B). In contrast, sample-based filters (frequency filter, Decontam frequency filter) were ineffective in reducing potential contaminants. None of the decontamination algorithms affected the relative abundance of the potential contaminant *Acinetobacter*.

Interestingly, the number of reduced reads strongly varied between the decontamination algorithms: While the frequency filter and MicrobIEM kept > 90% of reads, a larger number of reads were removed with other decontamination tools (Additional file 1: Supplementary Figure 8A). Specifically, SourceTracker removed 97% of the reads, which can be explained by its default rarefaction depth of 1000 reads per sample. Similarly, the reduction of ASVs strongly depended on the chosen decontamination algorithm: the strict frequency filters and SourceTracker reduced the number of kept ASVs up to < 3% (3–93% frequency filter) or < 1% (1–28%) depending on the chosen thresholds. In contrast, the other decontamination algorithms kept 75–99% of ASVs (Additional file 1: Supplementary Figure 8B).

### MicrobIEM – workflow

#### Parameter selection in MicrobIEM

In contrast to other decontamination tools, MicrobIEM provides an interactive graphical user interface and allows the user to directly assess the effect of the currently chosen filter parameters on the dataset.

**Fig. 5:**
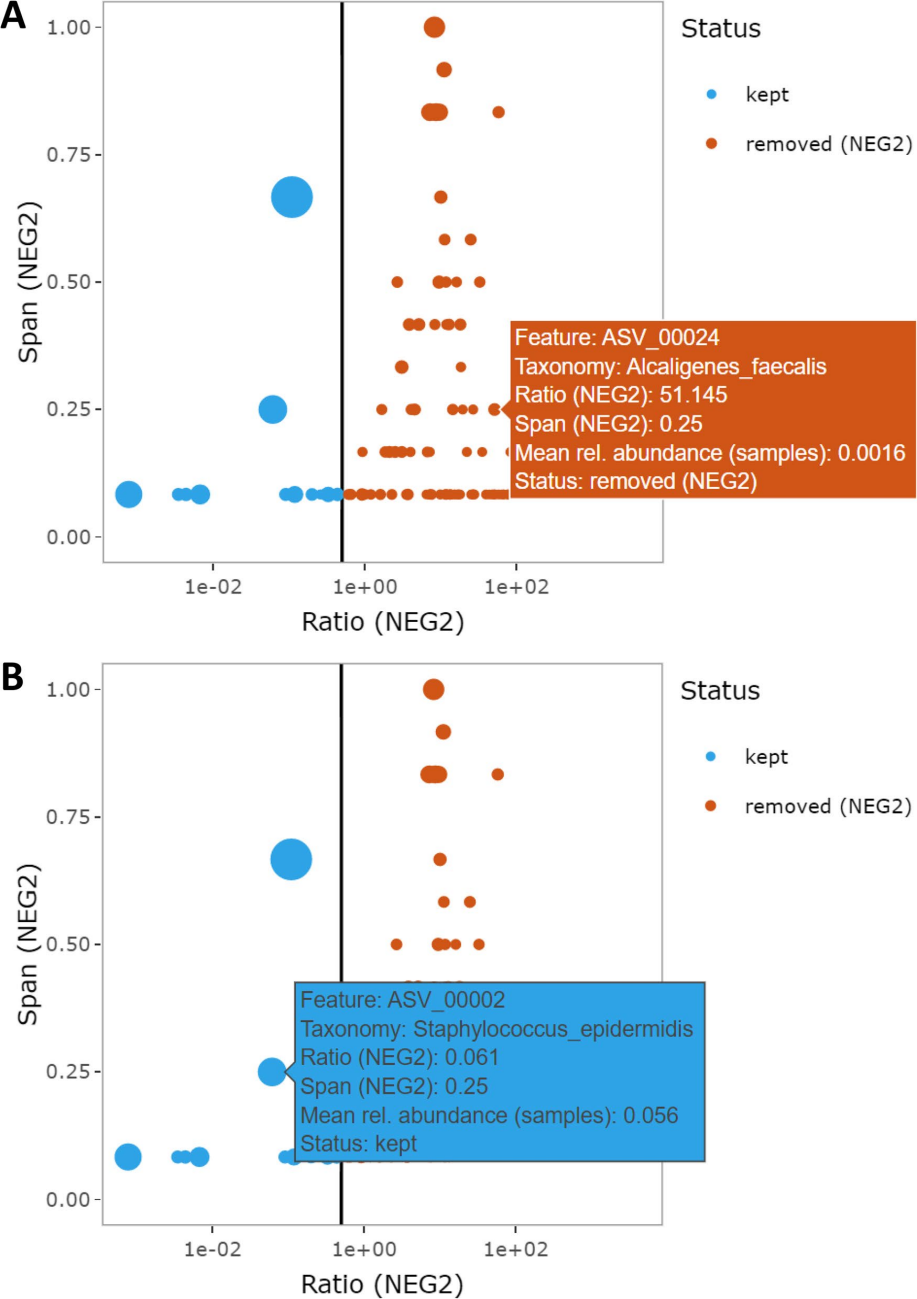
Screenshots of interactive graphical support for contamination removal with MicrobIEM. The interactive graphical user interface of MicrobIEM supports the user by displaying which features are removed (in orange, **A**) or kept (in blue, **B**) with the current filter threshold indicated as a vertical black line. In this example, filtering is based on the pipeline negative control NEG2. Each datapoint represents one feature (ASV or OTU) present in the selected control type (NEG2), and bubble area indicates the mean relative abundance per feature in the samples. Interactive hover texts (orange box in **A**, blue box in **B**) provide further information per feature, such as ID, taxonomy and mean relative abundance over samples

The effect of quality control choices on the data can be explored for each filtering parameter with the interactive figures provided in MicrobIEM (Fig. 5, Additional file 1: Supplementary Figure 9). Thus, users can monitor the reduction of reads per filter step (Additional file 1: Supplementary Figure 9A), visualize the number of reads and features per sample (Additional file 1: Supplementary Figure 9B), the ratio and span for contamination filtering (Fig. 5, Additional file 1: Supplementary Figure 9C) or track the loss of features over the whole quality control process (Additional file 1: Supplementary Figure 9D). Given the heterogeneity of microbiome data, the parameters chosen will depend on sample sequencing depth, available negative controls, and the amount of input material. Users can directly assess the impact of each filter step and decide on parameters based on the interactive graphics, showing useful information on, e.g., the abundance and taxonomic assignment of removed features. The available quality control steps in MicrobIEM can be either used or skipped (partially or altogether) to proceed directly to microbiome data analysis.

#### Analysis with MicrobIEM

**Fig. 6:**
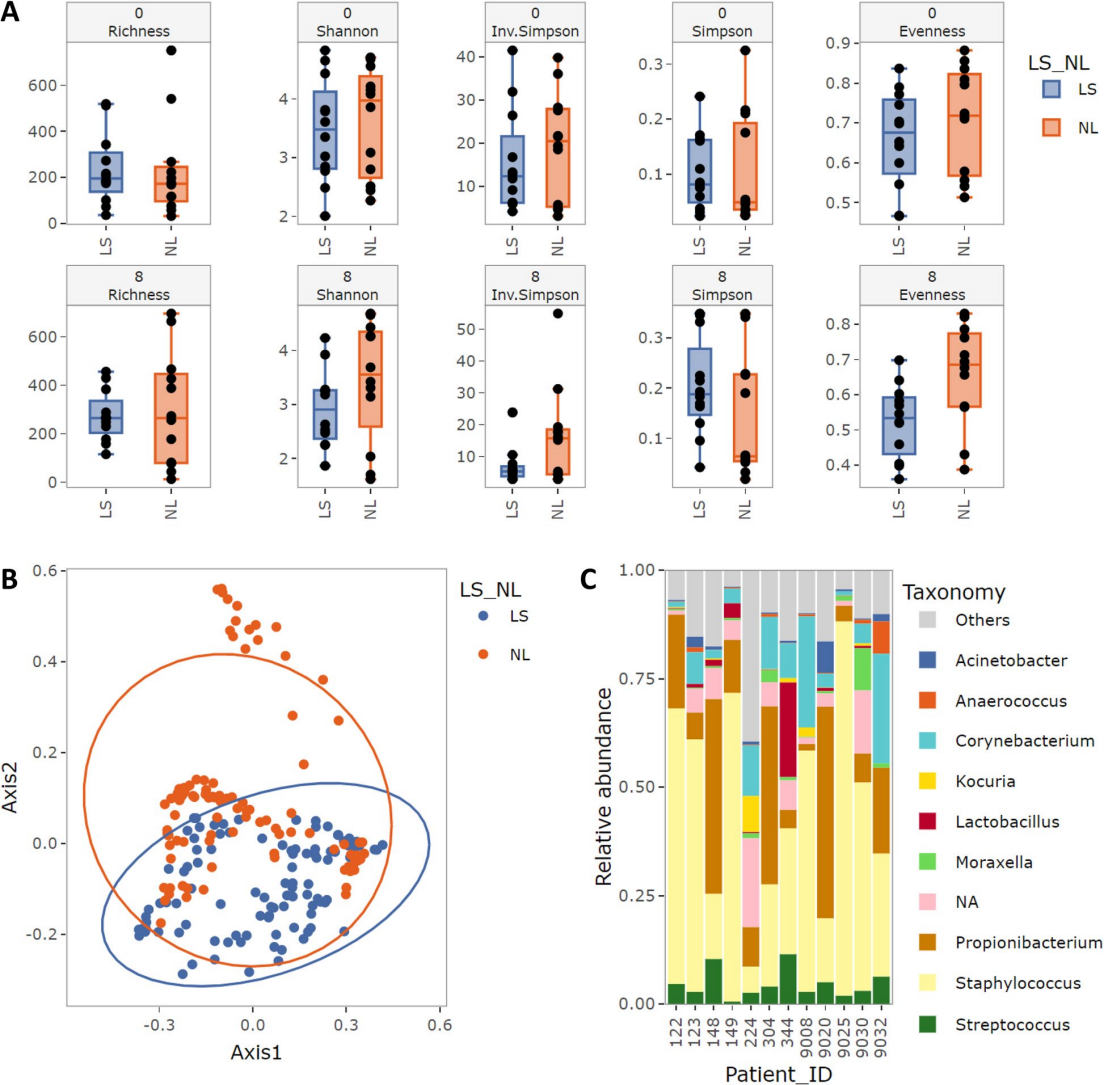
Screenshots of interactive graphical outputs from MicrobIEM's basic microbiome analysis options. The interactive graphical user interface of MicrobIEM facilitates basic microbiome analysis. Implemented are alpha diversity analysis (**A**), beta diversity analysis (**B**), and analysis of the taxonomic composition (**C**) based on metadata and an easy and dynamic sample selection within the tool. As an example, differences in microbiome alpha diversity at week 0 and week 8 (**A**) and in global microbiome structure (**B**) are shown by lesional (LS) versus non-lesional (NL) skin, while **C** displays the sample composition per patient on genus level at a selected timepoint (week 8). Dots in **A** and **B** indicate individual samples. Boxes in **A** denote the median and interquartile range (IQR, distance between 25 and 75th percentile), and whiskers represent values up to 1.5 times the IQR. Ellipses in **B** denote 95% confidence intervals around cluster centroids based on a multivariate t-distribution. Bars in **C** show the microbiome composition of the ten most abundant genera at one timepoint per patient, while remaining genera are summarized as “Others”

In addition to quality control, MicrobIEM allows basic microbiome analysis without any knowledge in coding, based on the provided metadata with, e.g., gender, health status, or treatment group. For all basic analysis, samples or groups can be conveniently in- or excluded dynamically without restarting the analysis. The user can explore differences in alpha diversity measures (Richness, Shannon, Inverse Simpson, Simpson, and Evenness), in beta diversity (principal coordinate analysis (PCoA), non-metric multidimensional scaling (nMDS)), and the sample composition on different taxonomic levels. As an example for the interactive data analysis options in MicrobIEM, the skin microbiome dataset is used. Alpha diversity between health status is stratified by time point (Fig. 6A). Figure 6B shows an example of beta diversity analysis, detecting differences in global microbiome composition between two study groups (healthy and AD), and Fig. 6C demonstrates an analysis of the microbiome composition per patient.

## Discussion

We benchmarked five established bioinformatic decontamination algorithms and the two filtering approaches of our novel tool MicrobIEM in three mock communities with differing biomass and structure. Additionally, we evaluated their effects on an environmental low-biomass dataset. While sample-based approaches achieved good results in an even mock community, control-based approaches performed better in the staggered mock communities. The Decontam prevalence filter and our novel MicrobIEM ratio filter specifically reduced contaminating reads while keeping expected reads, both in the staggered mock communities and in the low-biomass skin microbiome dataset. In contrast to other decontamination tools, MicrobIEM offers visual support for choosing an appropriate filtering threshold and basic microbiome analysis with an interactive graphical user interface suitable for non-bioinformaticians.

In particular, when working with low-biomass samples, contaminants are a major pitfall in microbiome research [16]. Our sequencing data of dilution series of even and staggered mock communities confirmed that lower biomass samples are characterized by increasing proportions of contaminants, while the concrete extent of contamination varied between datasets. Similarly, the 10 most abundant taxa in our low-biomass skin microbiome dataset also contained genera known as contaminants and not native to human skin (Additional file 1: Supplementary Table 3, 4 and 5). Concludingly, the impact of contaminants increases with decreasing biomass: whereas contaminants do not play a major role in high-biomass samples such as stool, low-biomass environments such as the skin, lung [62], or duodenum [63] require bioinformatic decontamination to draw valid biological conclusions.

To evaluate different bioinformatic decontamination algorithms, we used mock communities with known sequence composition. Moreover, we compared the influence of the microbiome structure on the success of the tools by using an even and two staggered mock communities. While a previous pioneer benchmarking of decontamination approaches provided invaluable insights to the microbiome research community [20], these analyses were only performed using an even mock community. Indeed, we observed remarkable differences in the algorithm’s performances between the community structures of the three mocks, in line with previous research investigating sequencing data of even and staggered mock communities [64]. Since environmental microbiomes are typically composed of both major and minor species [28–30], we consider our two staggered mock communities to be more representative of natural microbiome samples. Therefore, also our benchmarking results on the staggered mock communities are likely more representative of handling contaminants in environmental microbiome samples. Moreover, our results strongly promote the need for using staggered, realistic mock communities for any type of benchmarking of methods in the field of microbiome research.

In addition to using a representative data basis for benchmarking, we compared several evaluation measures for the success of the decontamination tools. Accuracy is frequently evaluated in binary classification problems with only two possible outcomes (such as “mock” and “contaminant”), both in microbiome research [20] and science in general [31]. However, accuracy fails for imbalanced classifications [31]: If no contaminants are removed, accuracy is identical to the proportion of contaminant reads in the data, which is considered as baseline accuracy. Consequently, high accuracy can be achieved even though a decontamination tool did not remove any contaminants. The frequently recommended MCC has a more intuitive interpretation, but still lacks comparability between studies and samples by incorporating class imbalance. Therefore, we focused on Youden’s index to evaluate the performance of the decontamination algorithms, since it considers sensitivity and specificity equally, without the internal flaws described for accuracy and MCC.

Taken together, a fair and realistic benchmarking requires representative data and an unbiased evaluation methodology, which is why we suggest using staggered mock communities and Youden’s index to benchmark bioinformatic decontamination approaches.

The three main approaches for bioinformatic contaminant removal are (i) based on a contaminant blacklist, (ii) sample-based, and (iii) control-based.

Removing all sequences based on a contaminant blacklist (i) is error-prone as the sampled environment is not considered. Taking our mock communities as an example, expected genera like *Pseudomonas* and *Enterococcus* would have been removed with the blacklist-based approach. This problem becomes even more drastic in our skin microbiome dataset: *Staphylococcus, Cutibacterium*, and other main skin genera would have been removed even though they are also expected genera on the skin. Hence, the blacklist approach was not further investigated in this benchmarking study. This large overlap between skin genera and contaminants can be partially explained by operator-sample transfer, which often leads to the detection of skin inhabitants as contaminants in other samples [23]. The overlap between skin and contaminating genera, as well as the absence of the known sample composition, challenged the evaluation of decontamination algorithms in our skin dataset. However, the investigation of air or surface microbiomes might be affected by similar problems. Taken together, our results support previous conclusions [20] that the blacklist approach has to be carried out with caution and knowledge about the target environment.

Sample-based decontamination approaches (ii) include the most commonly used frequency filter. This filter reduced spurious taxa and increased reproducibility in one study [45]. Indeed, features with low relative abundance often represent incorrect sequences that should be removed to keep only true sequences in the microbiome dataset. However, minor species can also have an important role in microbial communities. The frequency filter is not specifically addressing contaminants and can only remove low-abundant features, which can be errors or true and potentially important sequences, as in our staggered mock community. Furthermore, the frequency filter assumes that contaminants are only present in low relative abundance [20], which is not the case in environmental low-biomass samples. As shown in our strongly diluted mock samples, contaminants appear in high relative abundance and cannot be removed with this approach, which may lead to false biological conclusions. Therefore, it is not surprising that sample-based decontamination was effective in the even mock community and in samples with high-input material (as reported previously [20, 45]), where all mock taxa are expected in higher relative abundance than the threshold for contamination removal. In line with that, sample-based decontamination tools performed poorly in lower-input samples of the staggered mock communities. Similarly, the frequency filter did not remove observed typical contaminants from the top ten genera of the skin dataset.

Similar problems were observed for the Decontam frequency filter, which only successfully worked in the even mock community. Of note, the algorithm’s assumption of small proportions of contaminants per sample is not fulfilled in our low-biomass data [25], and the approach does not address the problem of artificial negative correlations between input material and feature relative abundance due to the compositional nature of microbiome data [65]. Moreover, the algorithm requires additional information on the total amount of input material, which may not always be available by default and has to be measured retrospectively as in our skin microbiome dataset. While different methods for quantifying DNA may affect the efficiency of the Decontam frequency filter, we did not find differences in decontamination performance between quantification methods (see Supp. Table 1), but only between mock community structure.

Taken together, we observed that the sample composition and biomass influence the success of the sample-based decontamination algorithms. Novel extensions of the frequency filter are, e.g., PERFect, which ranks a taxon’s importance and contribution to total covariance and offers data-driven significance thresholds, achieving good results in mock and environmental datasets [66].

Control-based decontamination algorithms (ii) were able to reduce contaminants in all mock communities but outperformed the sample-based measures only in the more realistic, staggered mock communities. However, these control-based decontamination algorithms are only as good as the available controls. Therefore, it is advisable to include several replicates of controls to track systematic contaminants, and to cover the whole experimental pipeline. PCR controls can only cover contaminants introduced from the amplification step on, and usually contain very low number of reads, which may originate from cross-contamination [67]. Therefore, suitable controls are pipeline negative controls, which are processed along with the samples and accumulate contaminants from each step in the workflow. These negative controls can be used to compare the amount of background bacterial DNA to that of a low-biomass environment (e.g., via qPCR), as was done in the controversial case of the placental microbiome [22, 68, 69], or to correct microbiome data for background contamination.

Nonetheless, removing all sequences appearing in the negative control, as done by the presence filter, is not advisable due to the possibility of high false-positive detection of contaminants due to the aforementioned cross-contamination between samples and barcode errors from the sequencing process [20, 70]. In our benchmarking, the presence filter achieved surprisingly good results, which can be explained by the rare presence of mock taxa in the negative controls of all mock datasets. However, in the skin dataset, we could clearly show that the presence filter strongly reduced the relative abundance of *Cutibacterium*, a well-documented skin inhabitant present here in the negative controls, potentially due to cross-contamination. Although we did not specifically address cross-contamination in our benchmarking, averaging over replicates in our staggered mock communities might compensate the bias of sporadic cross-contamination. Improvements of the simple presence filter aim to alleviate the problem of cross-contamination in negative controls, such as implemented in the Decontam prevalence filter, SourceTracker [20, 25, 27], and the ratio and span filter of our novel tool MicrobIEM. SourceTracker performed poorly in our datasets, but achieves excellent results when additional samples of the lab environment or contaminant profiles are available, to the cost of having very long runtimes [20]. These additional samples were not available in our study design, but might have significantly improved SourceTracker’s decontamination performance. In the case of cross-contamination of mock taxa into negative controls, as in our staggered mock B, MicrobIEM’s span filter indeed performed better than the presence filter. Interestingly, the combination of MicrobIEM’s ratio and span filter did not improve the individual filters’ performance. Taken together, the Decontam prevalence filter and the MicrobIEM ratio filter achieved similarly convincing decontamination results across the two staggered mocks and the skin dataset, but results depended on the chosen threshold.

Our data showed that the ideal thresholds depend on the biomass and structure of a sample, and significantly impact the success of each decontamination algorithm as shown in our benchmarking. Despite the substantial impact of the chosen filtering parameters, none of the established tools support the user to identify a suitable parameter for the dataset. An ideal solution would be an automated, data-driven selection of parameters for all available decontamination algorithms, which is not yet available. MicrobIEM offers the unique advantage of interactive plots to help users with parameter selection and to evaluate the impact of the current settings on the final dataset. In contrast to Decontam and SourceTracker, which demand some experience in coding from their users, MicrobIEM is available both as a standalone R function for users with coding skills, and as a complete tool with graphical user interface with short runtime. No additional information such as quantification data is required to start the microbiome analysis with MicrobIEM.

## Conclusions

With this study, we confirmed that low-biomass microbiome samples are susceptible to contamination, which strongly affect the sample composition. Computational elimination of contaminants is an essential step to improve the quality of low-biomass next-generation microbiome sequencing data. With an improved framework for benchmarking of decontamination tools, we found that their performance strongly depends on the biomass and structure of the samples, as well as on the chosen tool-specific parameters. Decontam prevalence filter and our novel control-based decontamination tool MicrobIEM, particularly its ratio filter, successfully reduced contaminants in staggered, low-biomass samples. MicrobIEM uniquely offers a graphical user interface to help with parameter choice and is thus a suitable decontamination tool, also for experts without coding experience.

### Supplementary Information

**Additional file 1:** **TabS1.** Overview of datasets for benchmarking of bioinformatic decontamination tools. **TabS2.** Overview of strain and growth information of bacterial taxa used for the staggered mock community A. **TabS3.** Overview of common contaminants based on a literature search. **TabS4.** Overview of publications used for classification of typical skin inhabitants. **TabS5.** Typical skin inhabitants with appearance in at least three out of seven publications. **FigS1.** Schematic overview of decontamination filters implemented in MicrobIEM. **FigS2.** Schematic overview of MicrobIEM workflow for quality control and microbiome analysis. **FigS3.** Benchmarking of MicrobIEM’s ratio and span filter in the staggered mock communities. **FigS4.** Additional evaluation measures in the decontamination benchmarking of mock communities. **FigS5.** Precision-recall curves for decontamination benchmarking of mock communities. **FigS6.** Effect of decontamination algorithms in the low-biomass skin microbiome dataset. **FigS7.** Effect of decontamination algorithms on the top 10 genera in the low-biomass skin microbiome dataset. **FigS8.** Reduction of reads and features in the low-biomass skin microbiome dataset by decontamination tools. **FigS9.** Screenshots of graphical support for additional quality control measures with MicrobIEM.

## Data Availability

MicrobIEM is available on Github (https://github.com/LuiseRauer/MicrobIEM). The processed sequencing data of the even mock community is available on Github (https://github.com/lakarstens/ControllingContaminants16S). The raw sequencing data of the remaining datasets have been deposited at the European Nucleotide Archive (ENA) under accession number PRJEB67797 for the staggered mock A (https://www.ebi.ac.uk/ena/browser/view/PRJEB67797), accession number PRJEB67827 for the staggered mock B (https://www.ebi.ac.uk/ena/browser/view/PRJEB67827), and accession number PRJEB37663 for the skin microbiome dataset (https://www.ebi.ac.uk/ena/browser/view/PRJEB37663). Data analysis scripts used in this manuscript are available on Github (https://github.com/LuiseRauer/Decontamination_benchmarking) for benchmarking of the mock communities. Data analysis scripts for benchmarking of the skin microbiome dataset are available on the Open Science Framework (OSF) platform (https://osf.io/yn9sa/).

## Abbreviations

AD: Atopic dermatitis
ASV: Amplicon sequence variant
MCC: Matthews correlation coefficient
nMDS: Non-metric multidimensional scaling
OTU: Operational taxonomic unit
PCoA: Principal coordinate analysis
PERMANOVA: Permutational multivariate analysis of variance
