# Supplementary material for: Benchmarking MicrobIEM – a user-friendly tool for decontamination of microbiome sequencing data
Source: BMC Biol. 2023 Nov 23;21:269. doi: 10.1186/s12915-023-01737-5 (PMC10666409; doi:10.1186/s12915-023-01737-5)
Supplement: Supplementary file 1 — Additional file 1: TabS1. Overview of datasets for benchmarking of bioinformatic decontamination tools. TabS2. Overview of strain and growth information of bacterial taxa used for the staggered mock community A. TabS3. Overview of common contaminants based on a literature search. TabS4. Overview of publications used for classification of typical skin inhabitants. TabS5. Typical skin inhabitants with appearance in at least three out of seven publications. FigS1. Schematic overview of decontamination filters implemented in MicrobIEM. FigS2. Schematic overview of MicrobIEM workflow for quality control and microbiome analysis. FigS3. Benchmarking of MicrobIEM’s ratio and span filter in the staggered mock communities. FigS4. Additional evaluation measures in the decontamination benchmarking of mock communities. FigS5. Precision-recall curves for decontamination benchmarking of mock communities. FigS6. Effect of decontamination algorithms in the low-biomass skin microbiome dataset. FigS7. Effect of decontamination algorithms on the top 10 genera in the low-biomass skin microbiome dataset. FigS8. Reduction of reads and features in the low-biomass skin microbiome dataset by decontamination tools. FigS9. Screenshots of graphical support for additional quality control measures with MicrobIEM. [file 12915_2023_1737_MOESM1_ESM.docx]

**Supplementary material**

**Benchmarking MicrobIEM – a user-friendly tool for decontamination of microbiome sequencing data**

Claudia Hülpüsch*^1,2,3^, claudia.huelpuesch@uni-a.de

Luise Rauer*^1,2,4^, luise.rauer@tum.de

Thomas Nussbaumer^4^, thomasnbionf@gmail.com

Vera Schwierzeck^4,5^, Vera.Schwierzeck@ukmuenster.de

Madhumita Bhattacharyya^1,2^, madhumita.bhattacharyya@tum.de

Veronika Erhart^1^, veri496@web.de

Claudia Traidl-Hoffmann^1,2,3,4,6^, claudia.traidl-hoffmann@med.uni-augsburg.de

Matthias Reiger^+1,2,4^, matthias.reiger@uni-a.de

Avidan U. Neumann^+1,4^, avidan.neumann@uni-a.de

^1^Environmental Medicine, Faculty of Medicine, University of Augsburg, Augsburg, Germany

^2^Chair of Environmental Medicine, Technical University of Munich, Munich, Germany

^3^CK CARE, Christine Kühne Center for Allergy Research and Education, Davos, Switzerland

^4^Institute of Environmental Medicine, Helmholtz Munich, Augsburg, Germany

^5^Institute of Hygiene, University Hospital Muenster, Muenster, Germany

^6^ZIEL - Institute for Food & Health, Technical University of Munich, Freising-Weihenstephan, Germany

*These authors contributed equally to the work.

^+^These authors contributed equally to the work.

# Supplementary Tables

**Supplementary table 1: Overview of datasets for benchmarking of bioinformatic decontamination tools.** The three mock communities and the environmental skin microbiome dataset used for the benchmarking cover a broad range of bacterial biomass.

| **Microbiome type** | **Mock** | **Mock** | **Mock** | **Environmental** |
| --- | --- | --- | --- | --- |
| **Name** | **Even mock community** | **Staggered mock community A** | **Staggered mock community B** | **Skin microbiome dataset** |
| **Reference** | Karstens et al. Controlling for Contaminants in Low-Biomass 16S rRNA Gene Sequencing Experiments. mSystems. 2019. doi: 10.1128/mSystems.00290-19. | This paper | Rauer & De Tomassi et al. Bioinformatic correction of bacterial morphology-based extraction bias and chimeras in microbiome sequencing data. bioRxiv. 2023. doi: https://doi.org/10.1101/2023.07.06.547990 | Hülpüsch & Tremmel et al. Skin pH-dependent Staphylococcus aureus abundance as predictor for increasing atopic dermatitis severity. Allergy. 2020. doi: 10.1111/all.14461. |
| **Composition** | even | staggered | staggered | NA |
| **Number of bacterial taxa** | 8 | 15 | 3 | NA |
| **Number of samples used in this paper (excluding negative controls)** | 9 | 26 | 8 | 209 |
| **Number of pipeline controls used in this paper** | 1 | 3 | 4 | 12 |
| **Number of PCR controls** | 0 | 3 | 2 | 0 |
| **Dilution steps** | 1:3 | 1:10 | 1:20 | NA |
| **Number of dilutions** | 9 | 8 | 2 | NA |
| **Number of dilutions used in benchmarking** | 4 | 4 | 2 | NA |
| **Number of replicates per dilution** | 1 | 3 | 4 | NA |
| **Theoretical range cell numbers used in benchmarking** | 1.7 x10^8^ - 2.3x10^5^ | 1 x10^8^ - 2.3x10^5^ | 1.1x10^5^ - 5.5x10^3^ | NA |
| **DNA quantification method** | NanoDrop spectrophotometer | NanoDrop spectrophotometer | quantitative PCR (qPCR) | quantitative PCR (qPCR) |
| **DNA range of samples used in benchmarking** | 13.8-105.8 ng/µl | 10.5-34.8 ng/µl | 451-13,777 16S gene copy numbers | 1.46x10^1^- 1.42x10^7^ 16S gene copy numbers |

**Supplementary table 2:** **Overview of strain and growth information of bacterial taxa used for the staggered mock community A.**

| **Strain information** | | | **Growth information** | | | |
| --- | --- | --- | --- | --- | --- | --- |
| **Strain** | **Gram stain** | **% cells** | **°C** | **Medium broth** | **Medium agar** | **Atmosphere** |
| ***Bacillus subtilis*** | Positive | 0.18 | 32 °C | PC bouillon | TSA | aerob |
| ***Escherichia coli*** | Negative | 0.18 | 32 °C | PC bouillon | TSA | aerob |
| ***Lactobacillus reuteri*** | Positive | 0.18 | 32 °C | MRS | TSA | anaerob |
| ***Staphylococcus hominis*** | Positive | 0.18 | 32 °C | PC bouillon | TSA | aerob |
| ***Streptococcus pneumoniae*** | Positive | 0.18 | 37 °C | PC bouillon | APT | anaerob |
| ***Acinetobacter baumannii*** | Negative | 1.80 | 32 °C | PC bouillon | TSA | aerob |
| ***Bacillus cereus*** | Positive | 1.80 | 32 °C | PC bouillon | TSA | aerob |
| ***Clostridium beijerinckii*** | Positive | 1.80 | 37 °C | PC bouillon | MRCM/DRCM | anaerob |
| ***Enterococcus faecalis*** | Positive | 1.80 | 32 °C | PC bouillon | TSA | aerob |
| ***Staphylococcus aureus*** | Positive | 1.80 | 32 °C | PC bouillon | TSA | aerob |
| ***Listeria monocytogenes*** | Positive | 18.02 | 32 °C | PC bouillon | TSA | aerob |
| ***Propionibacterium acnes***  ***(Cutibacterium acnes)*** | Positive | 18.02 | 32 °C | MRCM/DRCM | TSA | anaerob |
| ***Pseudomonas aeruginosa*** | Negative | 18.02 | 32 °C | PC bouillon | TSA | aerob |
| ***Staphylococcus epidermidis*** | Positive | 18.02 | 32 °C | PC bouillon | TSA | aerob |
| ***Streptococcus mutans*** | Positive | 18.02 | 32 °C | PC bouillon | TSA | aerob |

**Supplementary Table 3: Overview of common contaminants based on a literature search**. Contaminants were defined as in following papers: Barton, Taylor, Lubbers, & Pemberton, 2006; Ducarmon, Hornung, Geelen, Kuijper, & Zwittink, 2020; Eisenhofer et al., 2019; Glassing, Dowd, Galandiuk, Davis, & Chiodini, 2016; Grahn, Olofsson, Ellnebo-Svedlund, Monstein, & Jonasson, 2003; Lauder et al., 2016; Laurence, Hatzis, & Brash, 2014; Salter et al., 2014; Tanner, Goebel, Dojka, & Pace, 1998; Weyrich et al., 2019.

| Phylum | Frequency | Genus |
| --- | --- | --- |
| Actinobacteria | low | *Aeromicrobium*, *Arthrobacter*, *Atopobium*, *Beutenbergia*, *Brevibacterium*, *Curtobacterium*, *Dietzia*, *Geodermatophilus*, *Janibacter*, *Kocuria*, *Microbacterium*, *Micrococcus*, *Microlunatus*, *Patulibacter*, *Rhodococcus*, *Rothia*, *Tsukamurella* |
|  | moderate | *Actinomyces*, *Corynebacterium* |
|  | high | *Propionibacterium* |
| Bacteroidetes | low | *Capnocytophaga*, *Dyadobacter*, *Hydrotalea*, *Niastella*, *Olivibacter*, *Pedobacter*, *Porphyromonas*, *Sediminibacterium*, *Wautersiella* |
|  | moderate | *Chryseobacterium*, *Prevotella*, *Flavobacterium* |
| Deinococcus-Thermus | low | *Deinococcus* |
| Firmicutes | low | *Abiothrophia*, *Anaerococcus*, *Brevibacillus*, *Brochothrix*, *Clostridium*, *Coprococcus*, *Dialister*, *Facklamia*, *Geobacillus*, *Megasphaera*, *Staphylococcus*, *Veillonella* |
|  | moderate | *Bacillus*, *Enterococcus*, *Lactobacillus*, *Paenibacillus*, *Streptococcus* |
| Fusobacteria | moderate | *Fusobacterium*, *Leptotrichia* |
| Proteobacteria | low | *Achromobacter*, *Afipia*, *Aquabacterium*, *Asticcacaulis*, *Aurantimonas*, *Azoarcus*, *Azospira*, *Beijerinckia*, *Bosea*, *Brevundimonas*, *Caulobacter*, *Craurococcus*, *Curvibacter*, *Devosia*, *Duganella*, *Enterobacter*, *Hoeflea*, *Kingella*, *Leptothrix*, *Limnobacter*, *Mesorhizobium*, *Methylophilus*, *Methyloversatilis*, *Neisseria*, *Nevskia*, *Novosphingobium*, *Ochrobactrum*, *Oxalobacter*, *Paracoccus*, *Pedomicrobium*, *Polaromonas*, *Pseudoxanthomonas*, *Psychrobacter*, *Roseomonas*, *Schlegelella*, *Sphingobium*, *Sphingopyxis*, *Sulfuritalea*, *Undibacterium*, *Variovorax*, *Xanthomonas* |
|  | moderate | *Acidovorax*, *Acinetobacter*, *Bradyrhizobium*, *Burkholderia*, *Comamonas*, *Cupriavidus*, *Delftia*, *Enhydrobacter*, *Haemophilus*, *Herbaspirillum*, *Janthinobacterium*, *Massilia*, *Methylobacterium*, *Pelomonas*, *Phyllobacterium*, *Ralstonia*, *Rhizobium*, *Sphingomonas* |
|  | high | *Escherichia*, *Pseudomonas*, *Stenothrophomonas* |

**Supplementary Table 4:** **Overview of publications used for classification of typical skin inhabitants**

| **Short name** | **Article Type** | **Technique** |
| --- | --- | --- |
| Timm 2020 (Timm, Loomis et al. 2020) | Original | Cultivation |
| Perez Perez 2016 (Perez Perez, Gao et al. 2016) | Original | Sequencing |
| Byrd 2018 (Byrd, Belkaid et al. 2018) | Review | Sequencing |
| Khayyira 2020 (Khayyira, Rosdina et al. 2020) | Original | Cultivation and Sequencing |
| Saheb Kashaf 2022 (Saheb Kashaf, Proctor et al. 2022) | Original | Cultivation and Sequencing |
| Bewick 2019 (Bewick, Gurarie et al. 2019) | Original | Sequencing (re-analysis) |
| Ogai 2018 (Ogai, Nagase et al. 2018) | Original | Cultivation and Sequencing |

**Supplementary Table 5:** **Typical skin inhabitants with appearance in at least three out of seven publications**

| **Genus** | **Appearance in paper (n out of 7)** |
| --- | --- |
| *Corynebacterium* | 7 |
| *Staphylococcus* | 7 |
| *Kocuria* | 6 |
| *Micrococcus* | 6 |
| *Propionibacterium* | 6 |
| *Streptococcus* | 5 |
| *Paracoccus* | 5 |
| *Acinetobacter* | 4 |
| *Enhydrobacter* | 4 |
| *Pseudomonas* | 4 |
| *Anaerococcus* | 3 |
| *Bacillus* | 3 |
| *Lactobacillus* | 3 |
| *Microbacterium* | 3 |
| *Sphingomonas* | 3 |

# Supplementary Figures


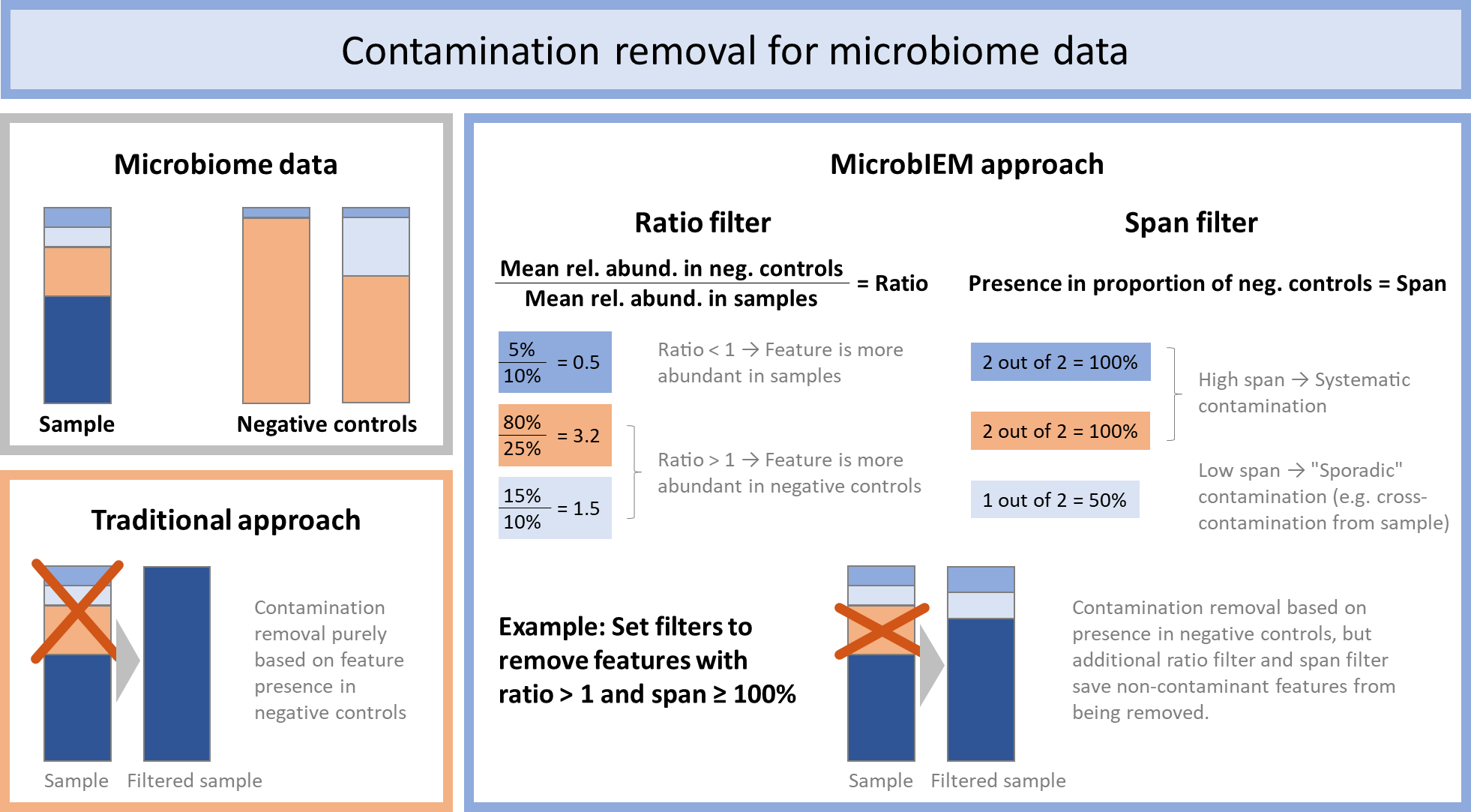


**Supplementary figure 1:** **Schematic overview of decontamination filters implemented in MicrobIEM.** MicrobIEM enhances the traditionally applied presence filter (removing all taxa appearing in negative controls) by implementing a ratio filter and a span filter, which aim to differentiate systematic contaminants from sporadic true reads present in negative controls, e.g. due to cross-contamination. Rel. abund. = relative abundance.


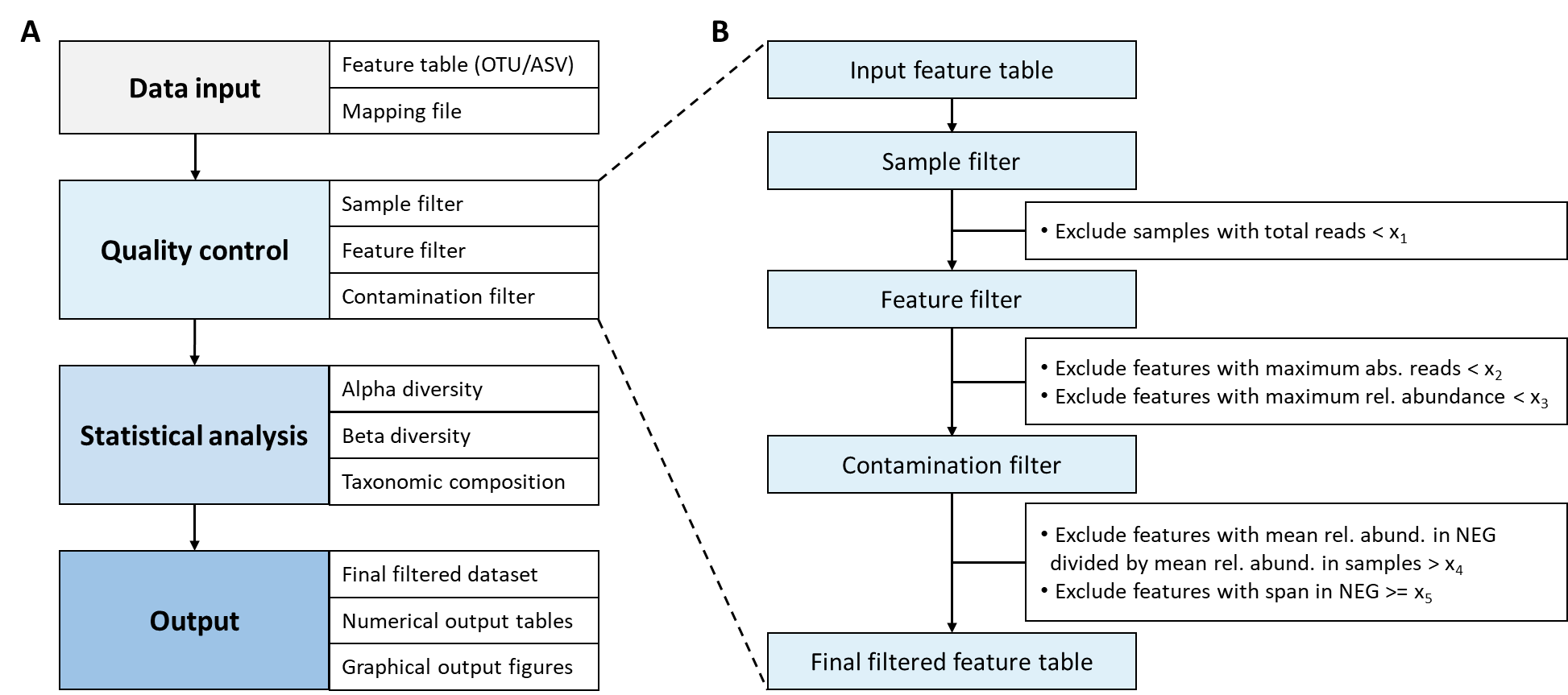


**Supplementary figure 2****: Schematic overview of MicrobIEM workflow for quality control and microbiome analysis.** (A) MicrobIEM requires a feature table with OTU or ASV counts and a mapping file as input, which are then interactively quality controlled and statistically analyzed. The final data and visualizations can be downloaded through the graphical user interface. (B) Quality control comprises sample and feature filters. First, shallow-sequenced samples can be visualized and removed. Next, spurious features can be identified by low read counts or low relative abundance, or by presence and relative abundance in negative controls compared to samples. The contamination filter is available for any two different types of negative controls. Abs. = absolute, rel. abund. = relative abundance.


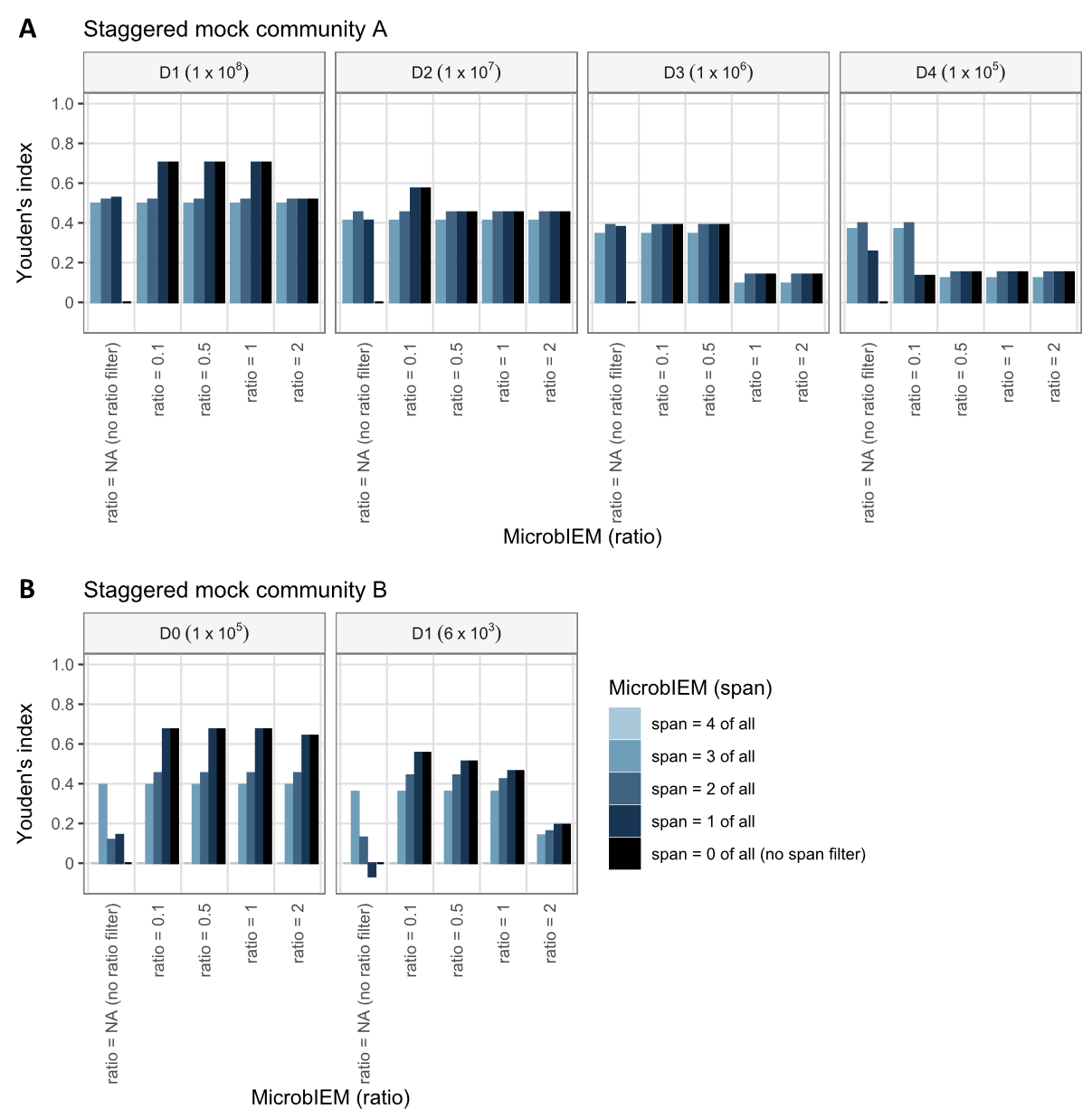


**Supplementary figure 3:** **Benchmarking of MicrobIEM’s ratio and span filter in the staggered mock communities**. Applying MicrobIEM’s span filter (2-4 of all) in the staggered mock A (A) or the staggered mock B (B) does not considerably improve the contaminant removal in these datasets. Only when no ratio filter (or a very strict ratio filter of 0.1 in A) is applied in very low biomass samples of the staggered mock A (D4, A) or in the staggered mock B (B), the additional span filter prevents the removal of mock reads present negative controls. MicrobIEM’s span filter of “1 of all” with no ratio filter is equivalent to the presence filter, and the number of available thresholds for MicrobIEM’s span filter depend on the number of negative controls per dataset (A: 3, B: 4 pipeline negative controls). Each filter combination was evaluated by its ability to distinguish expected mock reads from contaminating reads (defined by reads not matching expected sequences, see methods for details), from high (10^8^) to low-biomass samples (10^3^ bacterial cells). The performance per filter combination was quantified by Youden’s index, ranging from 1 (perfect classification) over 0 (random classification) to -1 (reverse labels). MicrobIEM’s algorithm was run separately per dilution, and values in A represent mean values over triplicates per dilution, values in B represent mean values over four replicates per dilution.


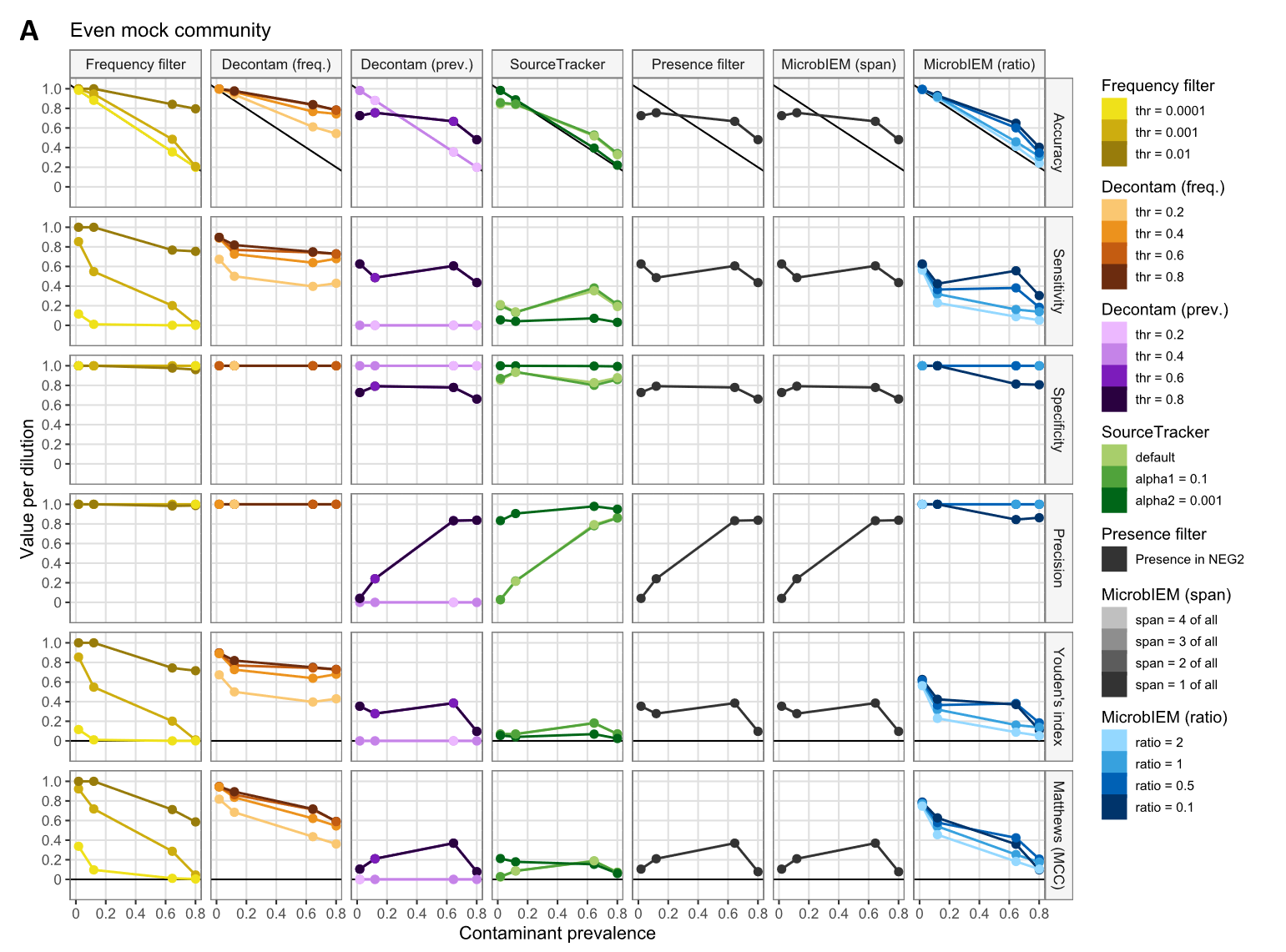

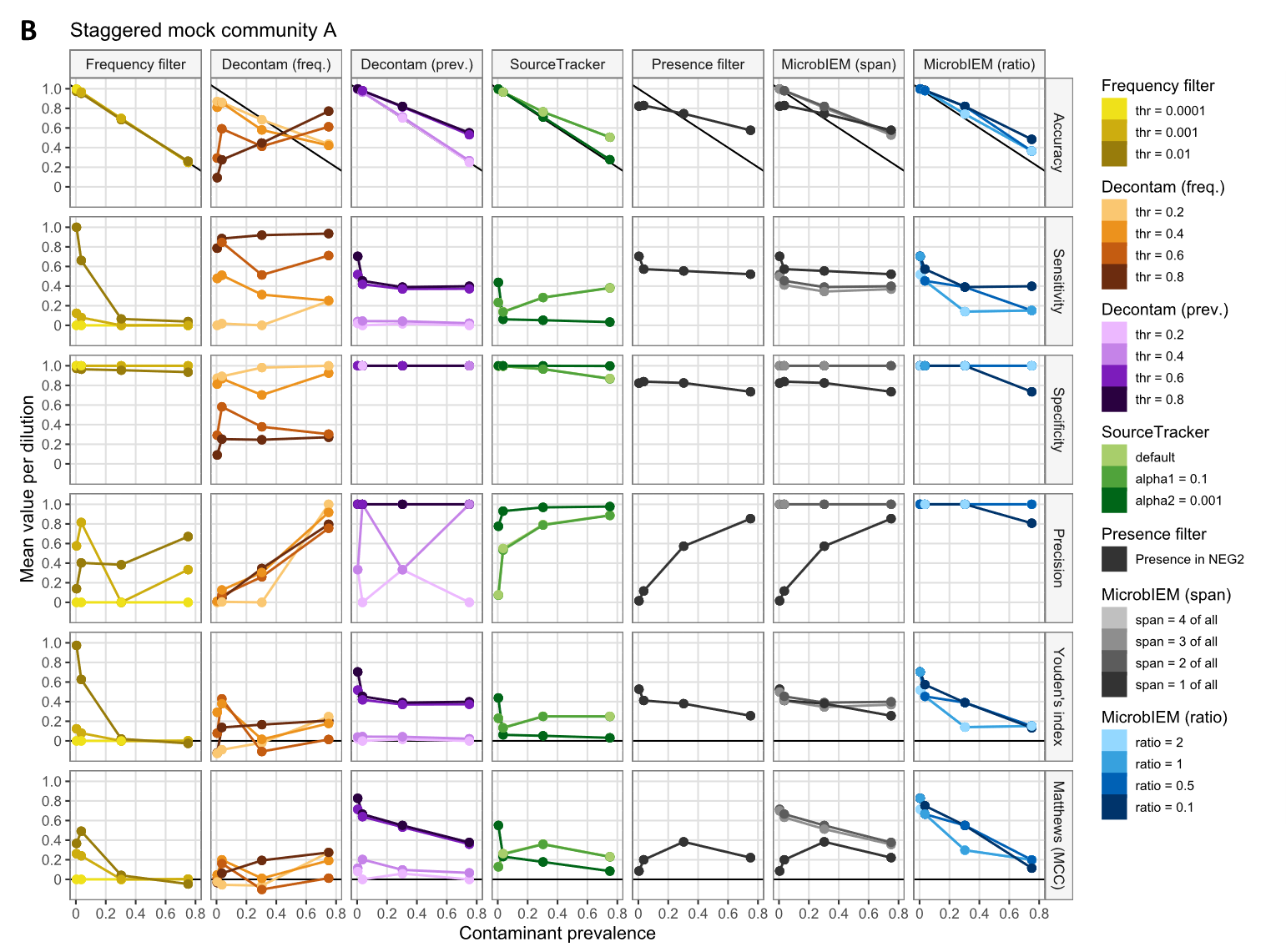


**
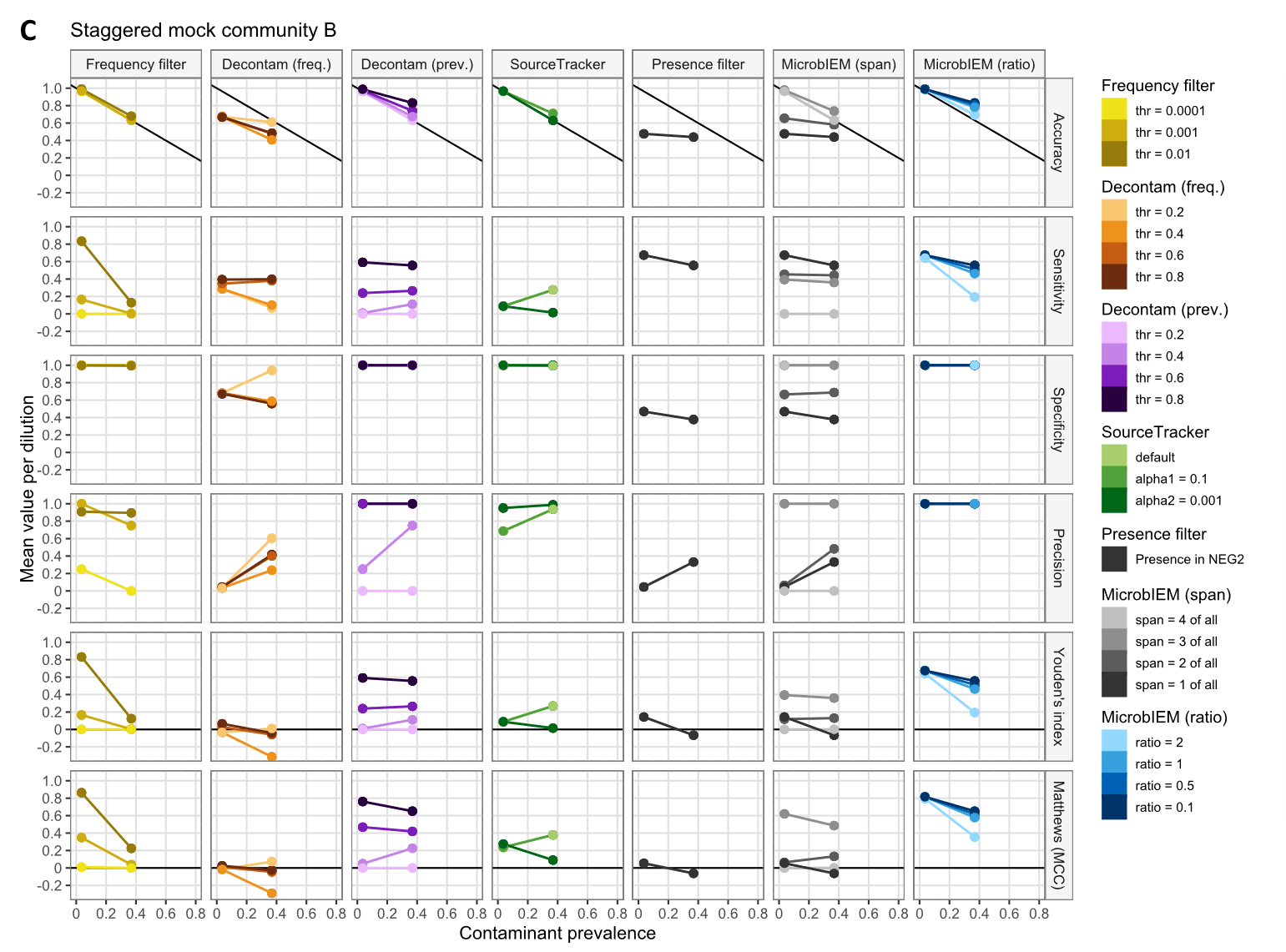
Supplementary figure 4:** **Additional evaluation measures in the decontamination benchmarking of mock communities**. Irrespective of the even (A) or staggered (B, C) mock community composition, the decontamination algorithms generally achieve higher specificity than sensitivity. Among the more comprehensive measures, accuracy needs to be compared to baseline accuracy (corresponding to contaminant prevalence per dilution, indicated by a diagonal black line), whereas MCC and Youden’s index present very similar results, and can be interpreted more intuitively to the horizontal black line at zero. Each algorithm was evaluated by its ability to distinguish expected mock reads from contaminating reads (defined by reads not matching expected sequences, see methods for details), from high (10^9^) to low-biomass samples (10^3^ bacterial cells). MicrobIEM’s span filter of “1 of all” is equivalent to the presence filter, and the number of available thresholds for MicrobIEM’s span filter depend on the number of negative controls per dataset (A: 1, B: 3, C: 4 pipeline negative controls). Algorithms were run separate per dilution, except for the Decontam frequency filter in (A) and SourceTracker in all datasets. Values in (B) represent mean values over triplicates per dilution, and values in (C) represent mean values over four replicates per dilution. Freq. = frequency, prev. = prevalence.


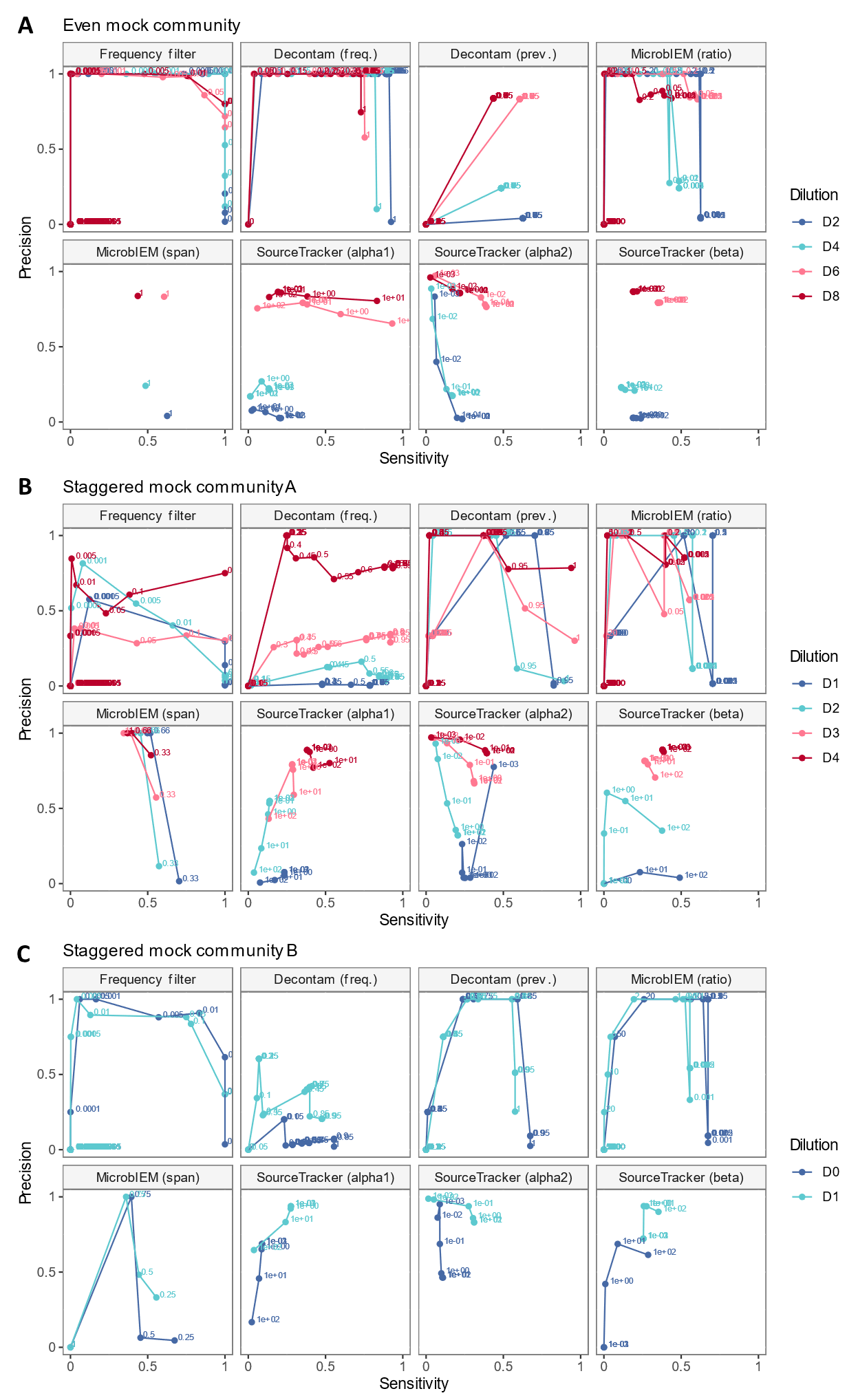


**Supplementary figure 5:** **Precision-recall curves for decontamination benchmarking of mock communities.** The performance of bioinformatic decontamination approaches varies greatly depending on the chosen thresholds per algorithm in the even mock community (A) and in the staggered mock communities A and B (B, C). Best possible results would be high sensitivity (also known as recall) in combination with high precision, and would appear in the upper right corner per panel. Each algorithm was evaluated by its ability to distinguish expected mock reads from contaminating reads (defined by reads not matching expected sequences, see methods for details), from high (10^9^) to low-biomass samples (10^3^ bacterial cells). MicrobIEM’s span filter of “1 of all” is equivalent to the presence filter. Algorithms were run separate per dilution, except for the Decontam frequency filter in (A) and SourceTracker in all datasets. Values in (B) represent mean values over triplicates per dilution, and values in (C) represent mean values over four replicates per dilution. Freq. = frequency, prev. = prevalence.


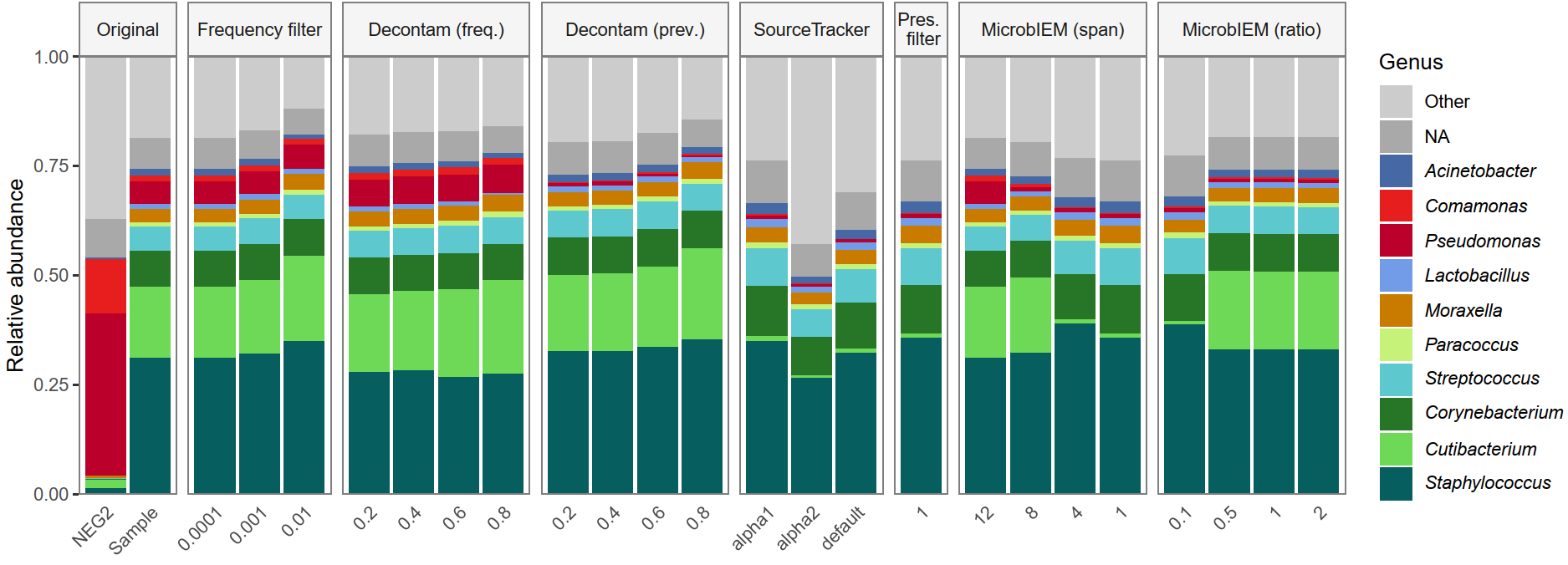


**Supplementary figure 6:** **Effect of decontamination algorithms in the low-biomass skin microbiome dataset.** The mean sample composition of the skin microbiome dataset (n=209 samples, 9 pipeline negative controls) is shown before filtering (original), and after applying sample-based and control-based decontamination tools with the different tool-specific thresholds. Per column, the mean sample composition of the top 10 genera appearing in the original dataset is displayed, while all remaining genera are summarized as “Others”. Pres. filter = presence filter, freq. = frequency, prev. = prevalence.

**
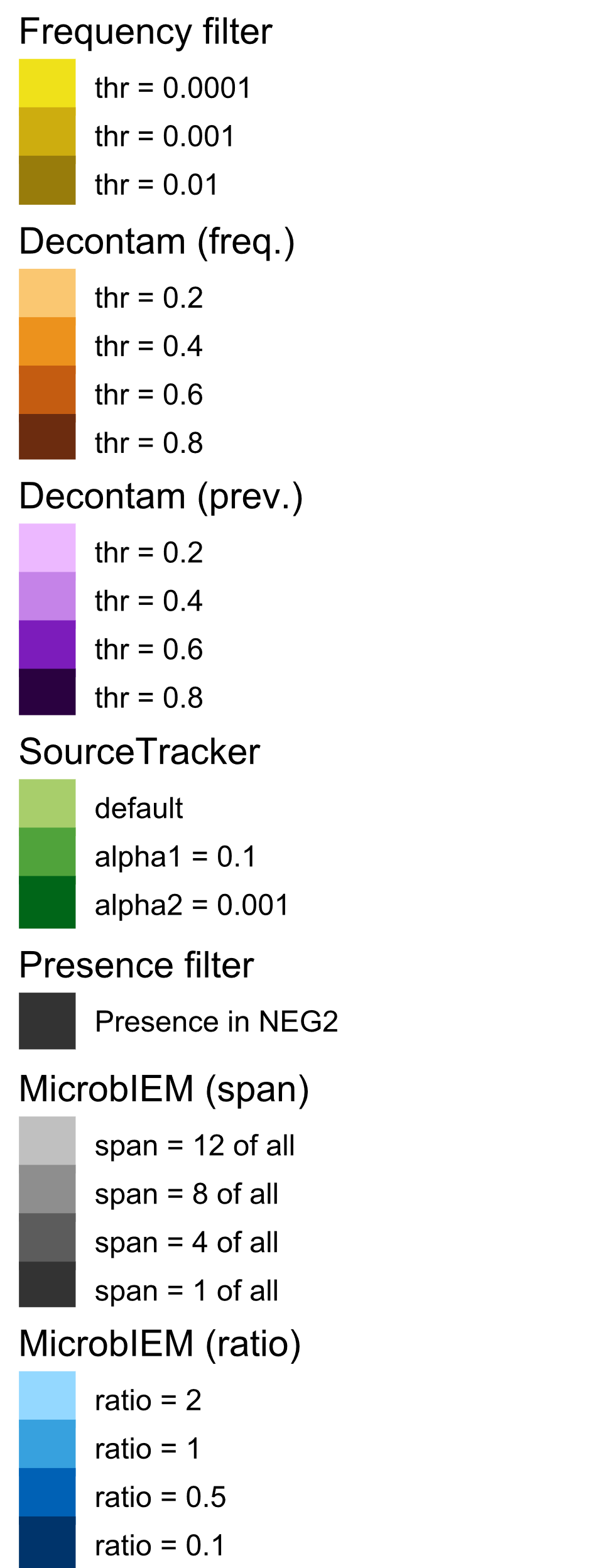
**
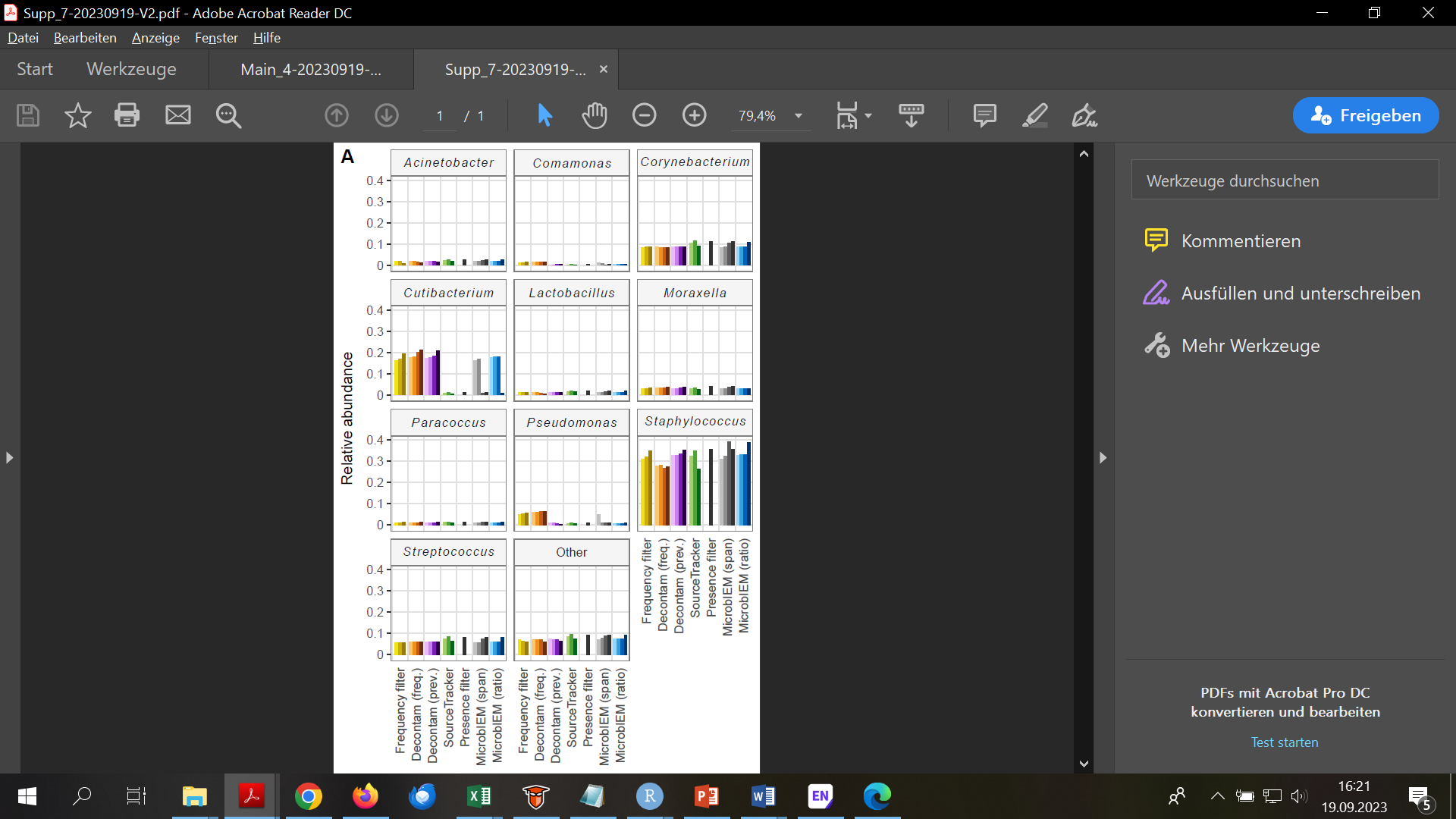


**Supplementary figure 7:** **Effect of decontamination algorithms on the top 10 genera in the low-biomass skin microbiome dataset.** While sample-based decontamination algorithms (Frequency filter, Decontam frequency filter) had little effect on the relative abundance of the top 10 genera of the low-biomass skin microbiome dataset, control-based decontamination algorithms (Decontam prevalence filter, SourceTracker, presence filter, MicrobIEM span filter, MicrobIEM ratio filter) specifically reduced *Pseudomonas* and *Comamonas*. MicrobIEM’s span filter of “1 of all” is equivalent to the presence filter. Freq. = frequency, prev. = prevalence.


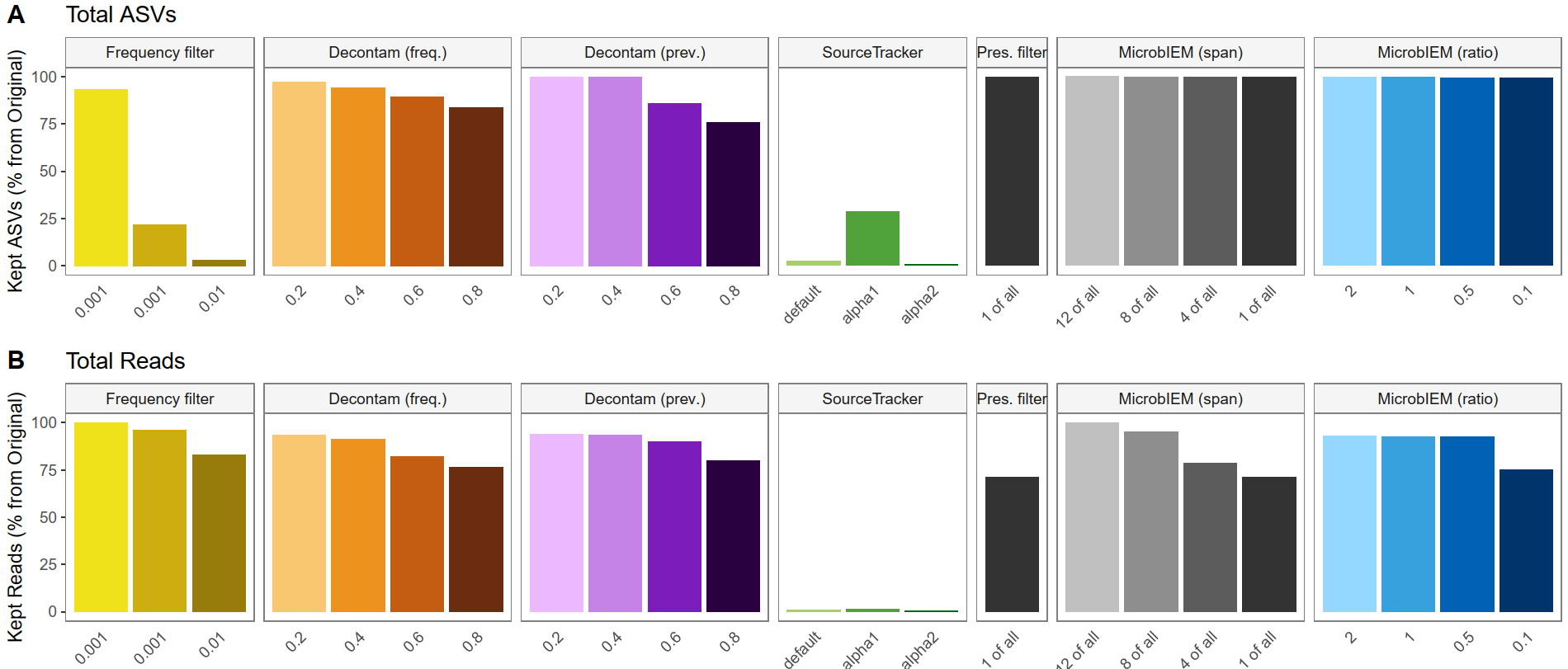


**Supplementary figure 8:** **Reduction of reads and features in the low-biomass skin microbiome dataset by decontamination tools.** The total reads (A) and ASVs (B) kept after the application of decontamination algorithms with different thresholds are shown in percent from the original number of reads, or ASVs, respectively, from the unfiltered low-biomass skin microbiome dataset (n=209 samples, 9 pipeline negative controls). Using a strict frequency filter or SourceTracker, > 90 % of ASVs were removed, while the other decontamination algorithms kept more ASVs. While SourceTracker removes > 95 % of reads, the other decontamination removed < 40 % of reads. Pres. filter = presence filter, freq. = frequency, prev. = prevalence.


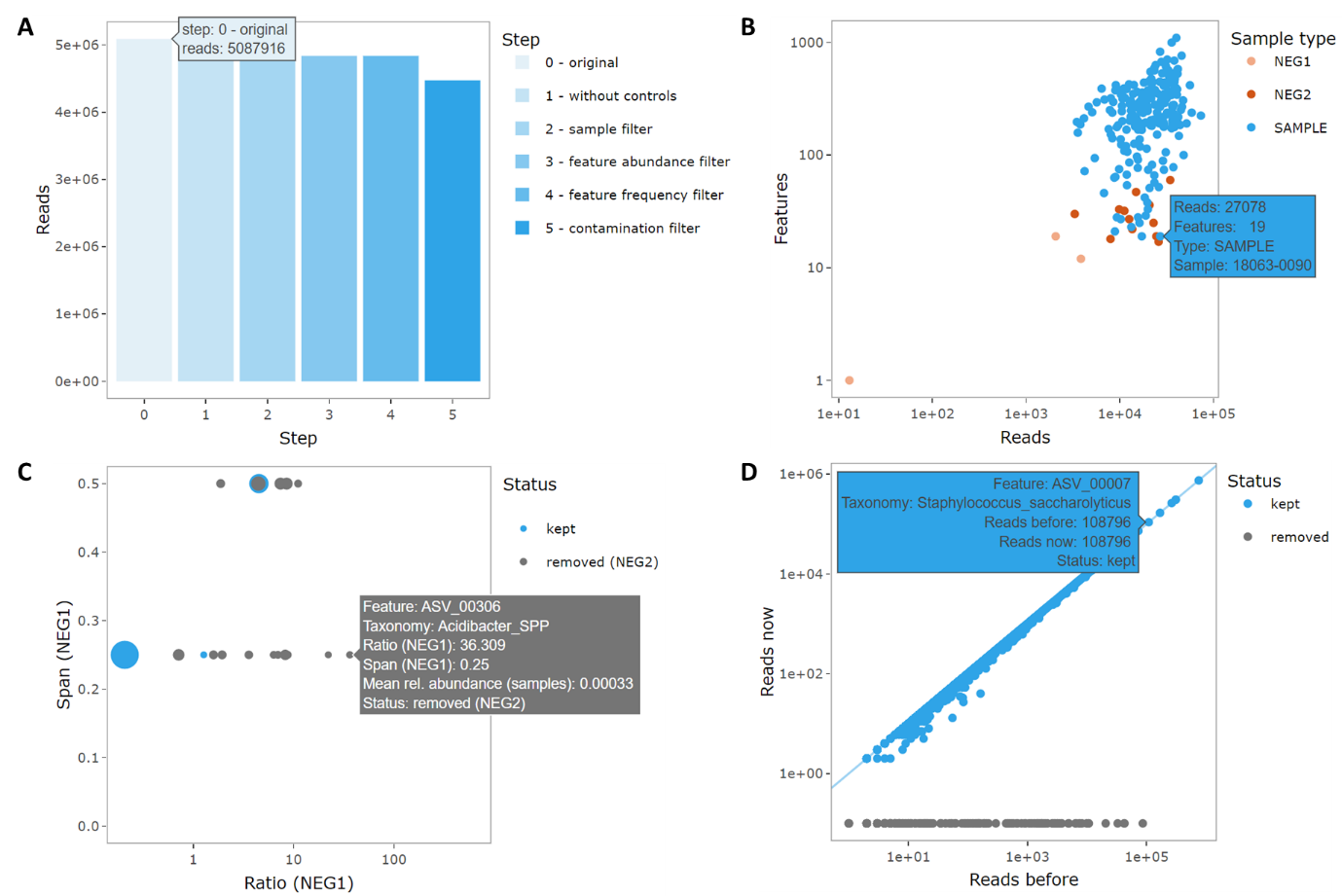


**Supplementary figure 9:** **Screenshots of graphical support for additional quality control measures with MicrobIEM.** MicrobIEM provides simple and interactive visual assessments of filtering criteria for microbiome data, including the reduction in the number of reads per filter step (A), the number of reads compared to the number of features per sample (B), taxa present in negative controls (shown for PCR negative controls NEG1) (C), and the number of reads per feature before and after applying the filtering steps (D). For each visualization implemented in MicrobIEM, hovering over a data point reveals further information, such as the number of reads and features per sample in B, or the ID and taxonomy per feature in D. Figure B displays features present in the NEG1 control, either in grey (removed by already applied NEG2 filter) or in blue (features not affected by current filter threshold). Since no NEG1 filter was applied here, no features appear in orange (features which would be removed by currently selected NEG1 filter threshold). Point size in C represents mean relative abundance of a feature in samples.

# References

Bewick, S., E. Gurarie, J. L. Weissman, J. Beattie, C. Davati, R. Flint, P. Thielen, F. Breitwieser, D. Karig and W. F. Fagan (2019). "Trait-based analysis of the human skin microbiome." Microbiome **7**(1): 101.

Byrd, A. L., Y. Belkaid and J. A. Segre (2018). "The human skin microbiome." Nature Reviews Microbiology **16**(3): 143-155.

Khayyira, A. S., A. E. Rosdina, M. I. Irianti and A. Malik (2020). "Simultaneous profiling and cultivation of the skin microbiome of healthy young adult skin for the development of therapeutic agents." Heliyon **6**(4).

Ogai, K., S. Nagase, K. Mukai, T. Iuchi, Y. Mori, M. Matsue, K. Sugitani, J. Sugama and S. Okamoto (2018). "A Comparison of Techniques for Collecting Skin Microbiome Samples: Swabbing Versus Tape-Stripping." Front Microbiol **9**: 2362.

Perez Perez, G. I., Z. Gao, R. Jourdain, J. Ramirez, F. Gany, C. Clavaud, J. Demaude, L. Breton and M. J. Blaser (2016). "Body Site Is a More Determinant Factor than Human Population Diversity in the Healthy Skin Microbiome." PLOS ONE **11**(4): e0151990.

Saheb Kashaf, S., D. M. Proctor, C. Deming, P. Saary, M. Hölzer, J. Mullikin, J. Thomas, A. Young, G. Bouffard, B. Barnabas, S. Brooks, J. Han, S.-l. Ho, J. Kim, R. Legaspi, Q. Maduro, H. Marfani, C. Montemayor, N. Riebow, K. Schandler, B. Schmidt, C. Sison, M. Stantripop, S. Black, M. Dekhtyar, C. Masiello, J. McDowell, M. Park, P. Thomas, M. Vemulapalli, M. E. Taylor, H. H. Kong, J. A. Segre, A. Almeida, R. D. Finn and N. C. S. Program (2022). "Integrating cultivation and metagenomics for a multi-kingdom view of skin microbiome diversity and functions." Nature Microbiology **7**(1): 169-179.

Timm, C. M., K. Loomis, W. Stone, T. Mehoke, B. Brensinger, M. Pellicore, P. P. A. Staniczenko, C. Charles, S. Nayak and D. K. Karig (2020). "Isolation and characterization of diverse microbial representatives from the human skin microbiome." Microbiome **8**(1): 58.
